# Dopamine suppresses pathological retinal oscillations and enhances the signal-to-noise ratio

**DOI:** 10.1073/pnas.2535572123

**Published:** 2026-10-06

**Authors:** B. Semihcan Sermet, Wouter Kamermans, Beerend H. J. Winkelman, Chris I. De Zeeuw, Maarten Kamermans

**Affiliations:** ^a^ Netherlands Institute for Neuroscience, Royal Netherlands Academy of Arts and Sciences, Amsterdam 1105 BA, The Netherlands; ^b^ Department of Neuroscience, Erasmus University Medical Center, Rotterdam 3000 DR, The Netherlands

**Keywords:** nystagmus, retina, amacrine cell, neuromodulation, dopamine

## Abstract

Infantile nystagmus is a debilitating involuntary eye movement disorder often associated with retinal diseases such as congenital stationary night blindness (CSNB). The oscillating eye movements of infantile nystagmus come with reduced visual acuity and strongly impair quality of life. No cure exists for this condition. Previously, we demonstrated that nystagmus in the CSNB mouse model *Nyx^nob^* has a retinal cause. Specifically, we found that synchronized oscillations of retinal ganglion cells (RGCs) are transmitted to the accessory optic system, triggering compensatory eye movements. The RGC oscillations appear to originate from a specific retinal cell type, the A_II_ amacrine cell (A_II_ AC), making these cells the preferred target for treatment. The reason for the oscillations in the A_II_ ACs is not fully understood. Here, we found that pharmacologically activating the D1 dopaminergic receptors on the A_II_ ACs in *Nyx^nob^* mice down-regulated their voltage-gated sodium and potassium channels, leading to a complete suppression of the pathological oscillations of both A_II_ ACs and RGCs. Furthermore, the signal fidelity of RGCs significantly improved due to the absence of the pathological oscillations. Our retinal network simulations confirm that the experimentally observed changes in properties of the A_II_ AC voltage-gated currents are necessary and sufficient to account for the dopamine-dependent abolishment of these oscillations. Our findings provide a mechanistic understanding of dopaminergic regulation of A_II_ ACs underlying infantile nystagmus.

## Significance

Oscillatory eye movements in infantile nystagmus associated with congenital stationary night blindness (CSNB) arise from abnormal retinal oscillations, yet the cellular basis of this instability has remained unclear. Here, we show that this oscillatory activity is strongly modulated by dopaminergic input to a specific retinal cell type, the A_II_ amacrine cell. In our mouse model for infantile nystagmus, retinal dopamine levels are reduced. Pharmacologically targeting D1 dopamine receptors blocks the oscillatory behavior of A_II_ amacrine cells and strongly improves the signal quality of retinal ganglion cells, the retina’s output neurons. As we previously showed that these oscillations drive infantile nystagmus, our study suggests a potential pharmacological therapeutic approach that targets retinal D1 receptors to suppress infantile nystagmus and improve visual performance.

One of the remarkable abilities of our brain is to stabilize images on the retina while moving around in our environment and, by doing so, facilitate perception. This task is accomplished by integrating signals from the retina and vestibular hair cells within the neural circuits controlling eye movements, including the accessory optic system (AOS) and the vestibulocerebellum (1). These systems, in turn, modulate the activity of abducens motor neurons, which drive the eye muscles. Multiple feedback loops and adaptive mechanisms act to minimize image motion over the retinal surface, i.e., retinal slip. When these mechanisms malfunction or are misaligned, nystagmus can be a consequence (2).

There are many forms of nystagmus (prevalence: approx. 0.2%) with many different causes. Nystagmus can be infantile or develop later in life, but in either case it is often accompanied by decreased visual acuity (3–6). This also holds for patients suffering from congenital stationary night blindness (CSNB), which is caused by genetic mutations affecting specifically synaptic transmission in the first synaptic layer of the retina; the synapse between their photoreceptor and ON-bipolar cells (ON-BC) is nonfunctional (7, 8). One of the proteins involved is the retina-specific protein nyctalopin (Nyx) (9). By analyzing the so-called *Nyx^nob^* mouse model, which expresses nonfunctional nyctalopin (10), we have been able to show that malfunction of the photoreceptor to ON-BC synapse makes retinal ganglion cells (RGCs) oscillate (11–13). This RGC population includes the ON direction-selective RGCs (dsRGCs). The ON-dsRGCs project to the AOS where they control eye movements, such as the optokinetic reflex (OKR). This was nicely illustrated by the work of Yonehara and colleagues (14), who showed that animals without ON-dsRGCs coding for horizontal motion do not have a horizontal OKR. In CSNB, the oscillating signals from the ON-dsRGCs are sent to the AOS, where they induce oscillatory eye movements, i.e., infantile nystagmus (13). However, the cellular mechanism underlying the RGC oscillations has not yet been fully resolved.

Previous work strongly implicates A_II_ amacrine cells (A_II_ ACs) as the source of this oscillatory firing of RGCs (12, 13). A_II_ ACs are highly connected neurons in the inner retina (*SI Appendix*, Fig. S1); they receive input from rod ON-BCs, are electrically coupled with each other as well as with cone ON-BCs, and they provide glycinergic inhibition to cone OFF-BCs, other ACs, and RGCs (15). In the dark-adapted retina, A_II_ ACs receive rod signals from the rod ON-BCs, amplify these signals by an active mechanism involving voltage-gated ion channels (I_Na_, I_K-A_, I_K-M_) (16), and transmit them to the ON- and OFF-pathways (17). In the light-adapted retina, the amplification by A_II_ ACs is not needed, because the large cone signals are transmitted directly from the BCs to the RGCs, suggesting that the properties of A_II_ ACs are modulated, most likely by dopamine, during light/dark adaptation.

The active amplification in the A_II_ ACs depends on the specific cellular arrangement of their ion channels, with I_Na_ and I_K-M_ being exclusively localized on a highly specialized process of the A_II_ AC, the initial segment (18), while I_K-A_ is present on the soma as well (19) (Fig. 2*A*). Under pathological circumstances, the amplification process can become unstable and lead to intrinsic oscillations, turning the A_II_ AC into an intrinsic oscillator (11, 20, 21). The oscillations can be explained by two possible reasons, which are not mutually exclusive. First, the oscillations appear when the membrane potential of A_II_ ACs is depolarized outside of its normal working range (11). This may well be the case in CSNB where the photoreceptor to rod ON-BC is nonfunctional and thereby leads to depolarization of the A_II_ AC membrane potential and induces oscillations. Second, it may be a dopamine-related problem. Dopamine (DA) levels change during the circadian cycle, with high DA levels in the light-adapted retina and low in the dark-adapted retina (22). A_II_ ACs receive a D1 dopaminergic input from dopaminergic amacrine cells (DAC) (18, 23–26), suggesting the activity of their voltage-gated ion channels may be modulated by DA. Indeed, Veruki and colleagues showed evidence of the dopaminergic downregulation of the sodium channels of A_II_ ACs in rats, leading to a shift in spike threshold (18). This implies that in the dark-adapted retina, low levels of DA allow for highly activated A_II_ AC sodium channels, leading to maximal amplification, enabling high-fidelity transmission of the rod signals. Conversely, in light-adapted conditions, there is no need for amplification of the large cone signals. The increased DA levels in the light-adapted retina may reduce the sensitivity of the sodium channels at the initial segment of the A_II_ ACs, minimizing the amplification.

In CSNB, DA release is reduced (27, 28), consistent with the notion that the activity of DACs is modulated by the rod-driven ON pathway (29, 30). Thus, we hypothesize that because of the low DA concentration in CSNB, the sodium channels on the initial segment (IS) of the A_II_ AC remain highly active in the light-adapted state and lead to spontaneous oscillations propagating in the retinal network and beyond, ultimately driving oscillating activity of RGCs and the oculomotor system downstream (12, 13). To test this hypothesis, we set out to investigate the impact of DA on the oscillations in the retinal network and the voltage-gated ion channels of A_II_ ACs in particular.

## Results

### DA Blocks RGC and A_II_ AC Oscillations.

**Fig. 1.**
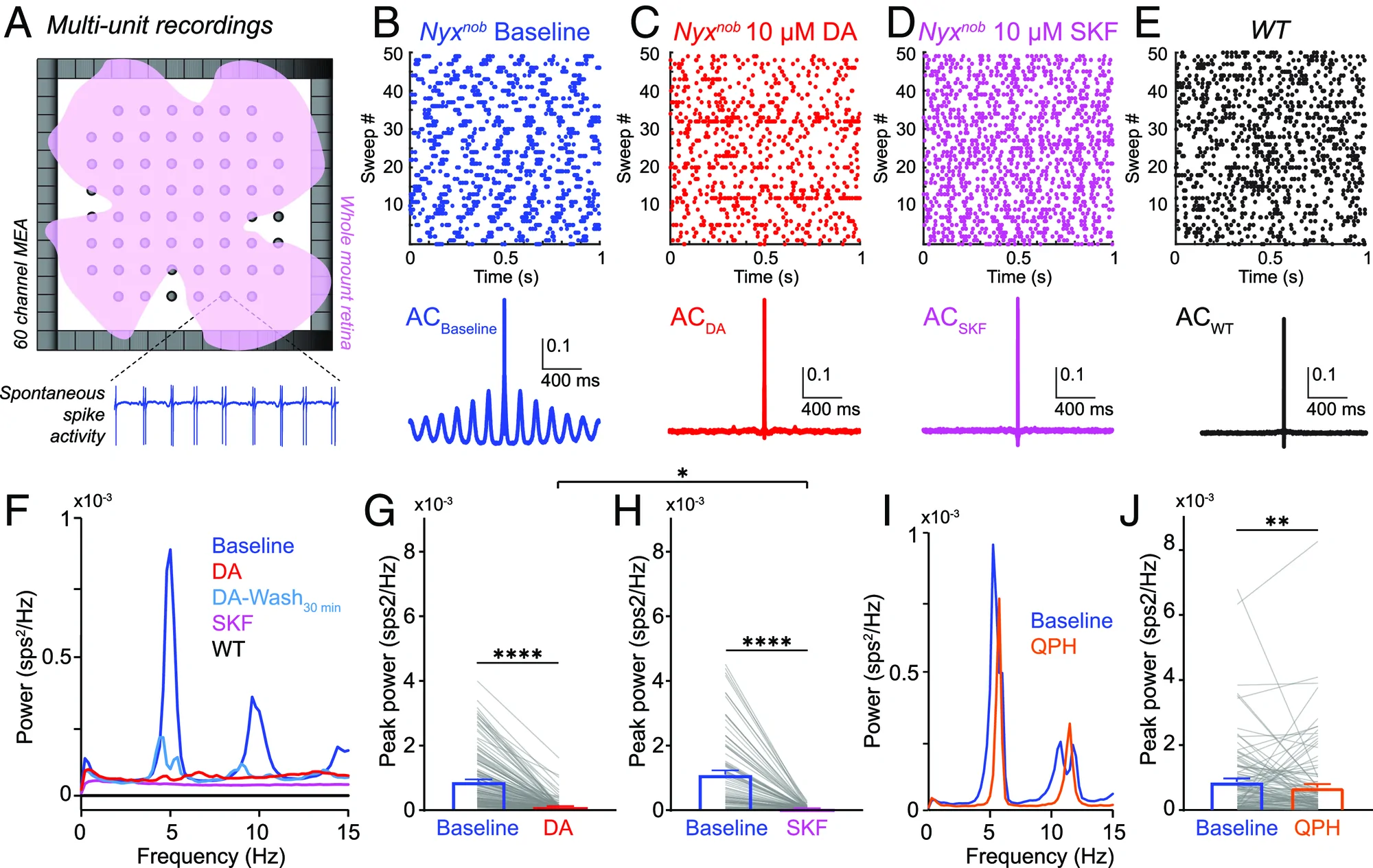
Dopamine suppresses RGC oscillations ex vivo in *Nyx^nob^* mice. (*A*) Schematic of whole-mount retina recording on a multielectrode array (MEA). (*B*–*E*) Spontaneous RGC activity in the dark in an isolated light-adapted retina. *Top*: raster plot of a single RGC. *Bottom*: autocorrelation of the same RGC. (*B*) Baseline *Nyx^nob^* mouse retina showing robust oscillations. (*C*) *Nyx^nob^* retina with 10 µM dopamine (DA) application. (*D*) *Nyx^nob^* retina with 10 µM D1 agonist SKF-81297 (SKF) application, showing complete suppression of oscillations. (*E*) Wild-type (WT) mouse retina showing no spontaneous oscillations. (*F*) Mean power spectral density of RGC activity from a representative retina under baseline conditions (blue), with DA (red), after DA washout (cyan), with SKF (magenta), and in a WT retina (black). (*G*) Paired comparison of baseline vs. DA oscillation power for individual RGCs (gray lines), with mean ± SEM overlaid (y-axis in sps^2^/Hz, sps = spikes per second). Baseline (blue, mean = 0.89 × 10^−3^ sps^2^/Hz, n = 165), DA (red, mean = 0.11 sps^2^/Hz × 10^−3^, n = 165). Baseline vs. DA, *P* < 0.0001 (Wilcoxon paired test). (*H*) Same analysis as in G, but for baseline (blue, mean = 1.23 × 10^−3^ sps^2^/Hz, n = 88) vs. SKF (magenta, mean = 0.06 × 10^−3^ sps^2^/Hz, n = 88). Baseline vs. SKF, *P* < 0.0001 (Wilcoxon paired test). DA vs. SKF, *P* = 0.013 (Mann–Whitney test). (*I*) Mean power spectral density of RGC activity from a representative cell under baseline conditions (blue) and with QPH (orange). (*J*) Same analysis as in *G* and *H*, but for baseline (blue, mean = 0.86 × 10^−3^ sps^2^/Hz, n = 122) vs. QPH (orange, mean = 0.69 × 10^−3^ sps^2^/Hz, n = 122). Baseline vs. QPH, *P* = 0.003 (Wilcoxon paired test).

To test the hypothesis that DA modulates the oscillations seen in RGCs, we recorded spontaneous RGC activity from isolated retinas of *Nyx^nob^* and wild-type (WT) mice using a multielectrode array (MEA). As shown by spike raster plots, Fourier transform, and autocorrelation analysis, many RGCs of *Nyx^nob^* mice, but not of WTs, exhibited robust spontaneous oscillations with a dominant frequency around 5 Hz (Fig. 1 *B, E*, and *F*). This result is similar to what we previously reported: Many RGCs, including the ON-dsRGCs, oscillate in Nyx^nob^ retinas (13). Application of 10 µM DA to the *Nyx^nob^* retina suppressed these oscillations (Fig. 1*C*). Quantitative analysis of mean power spectral density confirmed strong oscillations in baseline RGCs (mean 0.97 × 10^−3^ sps^2^/Hz, n = 253), which were significantly reduced by DA application (Fig. 1*G*; mean 0.11 × 10^−3^ sps^2^/Hz, n = 165; *P* < 0.0001, Wilcoxon matched-pairs signed-rank test), bringing the power of the dominant frequency to a level comparable to that of WT. Moreover, the DA effect was reversible in that a washout for 30 mins allowed the oscillations to reoccur (Fig. 1*F*).

To identify the type of DA receptor mediating this suppression, we tested the specific D1 DA agonist SKF-81297. Application of 10 µM SKF-81297 was also highly effective in suppressing these oscillations (Fig. 1 *D, F*, and *H*), reducing mean power to 0.06 × 10^−3^ sps^2^/Hz (n = 88 RGCs; *P* < 0.0001 vs. baseline, Wilcoxon matched-pairs signed-rank test). The efficacy of SKF-81297 was very high, indicated by a statistically significant difference when compared directly to DA (*P* = 0.0126, DA vs. SKF, Mann–Whitney test). In contrast, application of the D2-agonist quinpirole hydrochloride (QPH, 10 µM) hardly affected the RGC oscillations (Fig. 1 *I* and *J* and *SI Appendix*, Fig. S2), indicating that D1 receptor activation is the primary mechanism for suppressing the oscillations.

**Fig. 2.**
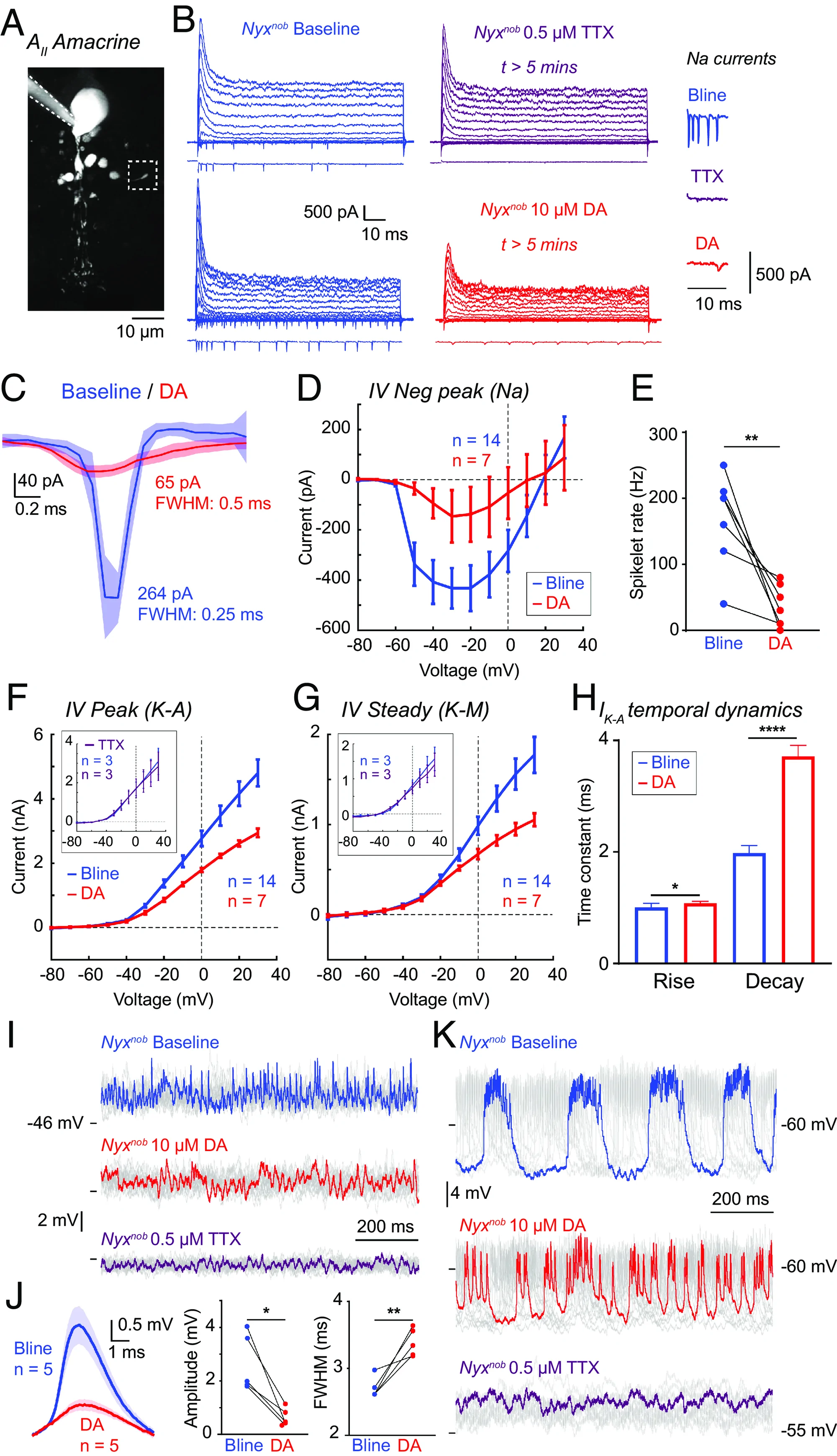
Dopamine modulates voltage-gated currents and suppresses oscillations in *Nyx^nob^* A_II_ amacrine cells. (*A*) Two-photon z-stack of a Lucifer Yellow-filled A_II_ AC in a retinal slice during whole-cell recording. The initial segment is highlighted with a white dashed square. (*B*) Representative voltage-clamp currents from a *Nyx^nob^* A_II_ AC in response to voltage steps (from −80 mV to +40 mV in 10 mV increments). Traces show baseline (*Top* and *Bottom Left*), after 0.5 µM TTX (*Top Middle*), and after 10 µM dopamine (DA) application (*Bottom Middle*). *Right*: zoomed-in transient inward currents I_Na_ at −50 mV. (*C*) Dopaminergic modulation of I_Na_ and spikelet kinetics. Isolated mean I_Na_ traces from a representative A_II_ AC in baseline (blue) and with DA (red). (*D*) Mean I–V for negative peak (Neg peak − I_Na_) in baseline (blue) and DA (red) conditions. (*E*) Quantification of spikelet rate at −50 mV (n = 7). DA significantly reduces spikelet rate (baseline: 168.5 Hz vs. DA: 35.7 Hz; *P* = 0.005, paired *t* test). (*F*) Mean I–V relationships for estimated potassium currents. *Left*: Peak outward current (0 to 10 ms after stimulus onset, mainly I_K−A_) in baseline (blue) and DA (red) conditions, showing DA-induced reduction. (*G*) Sustained outward current (80 to 100 ms after stimulus onset, mainly I_K-M_), in baseline (blue) and DA (red) conditions, no significant DA effect in physiological range. *Insets*: I–V relationships after 0.5 µM TTX (n = 3), showing no effect on potassium conductances. (*H*) Bar graphs of I_K−A_ temporal dynamics. Rise times: baseline (blue; mean 1.00 ms, n = 14) vs. DA (red; mean 1.08 ms, n = 7; *P* = 0.016, Mann–Whitney test). Decay time constants: baseline (blue; mean 1.98 ms, n = 14) versus DA (red; mean 3.71 ms, n = 7; *P* < 0.0001, Mann–Whitney test). DA significantly alters both rise and decay kinetics of I_K-A_. (*I*) Current-clamp traces recorded at resting membrane potential showing spikelets in baseline (*Top*), DA (*Middle*), and TTX (*Bottom*). (*J*) Quantification of spikelet modulation recorded at resting membrane potential (n = 5). *Left*: Isolated mean spikelet traces. *Right*: DA significantly reduces spikelet amplitude (baseline: 2.66 mV vs. DA: 0.65 mV; *P* = 0.012, paired *t* test) and increases Full Width at Half Maximum (FWHM; baseline: 2.71 ms vs. DA: 3.39 ms; *P* = 0.0078, paired *t* test), indicating suppressed and slowed I_Na_ kinetics. (*K*) Representative traces of unmasked oscillatory behavior in response to −30 pA current injection. Baseline cells show robust ~5 Hz oscillations (*Top*). DA disrupts the rhythm, resulting in irregular firing (*Middle*), while TTX completely abolishes all oscillatory activity (*Bottom*). All summary data are presented as mean ± SEM.

Since the RGC oscillations appear to originate from A_II_ ACs (12, 13), we next performed whole-cell patch-clamp recordings from A_II_ ACs (Fig. 2*A*) in light-adapted retinal slices from *Nyx^nob^* mice. The oscillations in A_II_ ACs in the retinitis pigmentosa mouse model rd1 are induced by the interaction of a fast potassium current (I_K-A_), a slow activating potassium current (I_K-M_), and a fast activating sodium current (I_Na_) (21). We therefore first considered whether the origin of the oscillations in CSNB was the same as in the rd1 mouse model. For that, we tested whether I_Na_ was indeed involved in generating the oscillations. In the Nyx^nob^ retinas, application of 0.5 µM TTX abolished the oscillations completely (Fig. 2*K*). In addition, the spikelets visible in the voltage clamp recordings were abolished as well (Fig. 2 *B* and *C*). The fact that we were unable to voltage clamp these spikelets suggests that they are initiated at the initial segment, a compartment connected to the soma via a high-resistance cable-like structure (18). These experiments show that the oscillations of A_II_ ACs in CSNB depend on the activation of I_Na_.

Next, we tested the effect of DA on the properties of A_II_ ACs. Application of 10 µM DA did not significantly change their resting membrane potential (*SI Appendix*, Fig. S3). To understand the ion channel basis of the dopamine effect on the A_II_ ACs, we examined voltage-gated currents using voltage-clamp recordings from A_II_ ACs. Voltage steps elicited large transient outward, transient inward, and sustained currents in baseline *Nyx^nob^* A_II_ ACs (Fig. 2*B*). The transient outward and inward currents most likely consist of the A-type potassium (I_K-A_) and a sodium current (I_Na_), respectively (21, 31). The sustained currents may reflect a combination of the I_K-A_ and M-type potassium (I_K-M_) currents. Notably, at a clamp potential of −50 mV, many unclamped spike-like events (spikelets) were observed (Fig. 2 *B* and *C*). Moreover, given that the voltage-gated channels driving these events are located at a distal initiation site, these recordings are subject to space-clamp limitations. Consequently, our measurements provide only rough estimates of the “real” IV-relation of I_Na_. Nevertheless, we constructed leak subtracted IV relations to get an estimate of the voltage dependence of this current (Fig. 2 *C* and *D*). I_Na_ is small and highly variable between cells. Given this large variability in the estimate of I_Na_, claims of the modulation of amplitude of I_Na_ cannot be reliably made.

Application of 10 µM DA visibly reduced the amplitude of I_K-A_ and I_Na_ transients (Fig. 2 *B* and *C*). Current-voltage (IV) relationships confirmed a significant reduction in the peak amplitude of I_K-A_ by DA, while the sustained I_K-M_ current remained largely unaffected in the physiological membrane potential range (Fig. 2*F*). To confirm specificity of our pharmacological targets, we applied 0.5 µM TTX; notably, TTX had no effect on the IV relationships for either I_K-A_ or I_K-M_ (Fig. 2 *F* and *G*, *Insets*), confirming that the I_Na_ could be isolated without interfering with potassium conductances. Kinetic analysis of I_K-A_ (Fig. 2*H*) revealed that DA significantly increased the rise time (baseline: 1.00 ms, n = 14 cells vs. DA: 1.08 ms, n = 7 cells; *P* = 0.0156, Mann–Whitney test) and nearly doubled the decay time constant (baseline: 1.98 ms, n = 14 cells vs. DA: 3.71 ms, n = 7 cells; *P* < 0.0001, Mann–Whitney test). The spikelet rate at −50 mV was 168.5 Hz and 35.7 Hz in baseline and DA conditions, respectively (n = 7 cells; *P* = 0.005, paired t test, Fig. 2*E*).

DA had a similarly profound effect on sodium-dependent activity. To quantify this modulatory effect of DA, we analyzed isolated spikelet events in current-clamp recordings at resting membrane potential (Fig. 2 *I* and *J*). DA application significantly reduced spikelet amplitude (baseline: 2.66 mV, n = 5 cells vs. DA: 0.65 mV, n = 5 cells; *P* = 0.012, paired *t* test) and increased the Full Width at Half Maximum (FWHM) (baseline: 2.71 ms, n = 5 cells vs. DA: 3.39 ms, n = 5 cells; *P* = 0.0078, paired *t* test), demonstrating both a reduction in I_Na_ magnitude and slowing of its kinetics.

Finally, we tested to what extent this modulation has an impact on the intrinsic oscillations of *Nyx^nob^* A_II_ ACs. To unmask their oscillatory potential, we utilized a steady hyperpolarizing current injection. Under these conditions, baseline cells display robust membrane potential oscillations (~5 Hz) with superimposed spikelets (Fig. 2*K*). In the presence of 10 µM DA, these rhythmic oscillations were blocked, while some irregular firing behavior persisted (Fig. 2*K*). In contrast, 0.5 µM TTX completely eliminated all oscillatory activity and spikelets (Fig. 2*K*). Together, these experiments show that DA modulates the ion channels that constitute the intrinsic oscillator in the A_II_ ACs. When D1 receptors are not activated, A_II_ ACs in *Nyx^nob^* can oscillate, whereas when they are actively engaged, the oscillations are suppressed.

### A_II_ AC Ion Channel Modulation Is Sufficient to Block Oscillations.

Since DA might also have modulated other retinal cell types (22), we wanted to assess in a computational model of A_II_ ACs (11) to what extent the measured DA-induced changes found in the A_II_ ACs are necessary and sufficient to account for the observed changes in the oscillatory behavior. Moreover, traveling through parameter space with the model may provide a better understanding of which changes at the level of the ion channels may lead to changes in oscillatory behavior. Since we cannot experimentally assess the ion currents in the IS, we used the model to determine what the changes were in the ion channel properties. Note that in principle, the amplitude of the spikelets may be reduced when the conductance of I_Na_ is lower, but also when I_Na_ slows down while I_K_ increases. Due to the space clamp problem, we cannot distinguish between the two conditions experimentally.

**Fig. 3.**
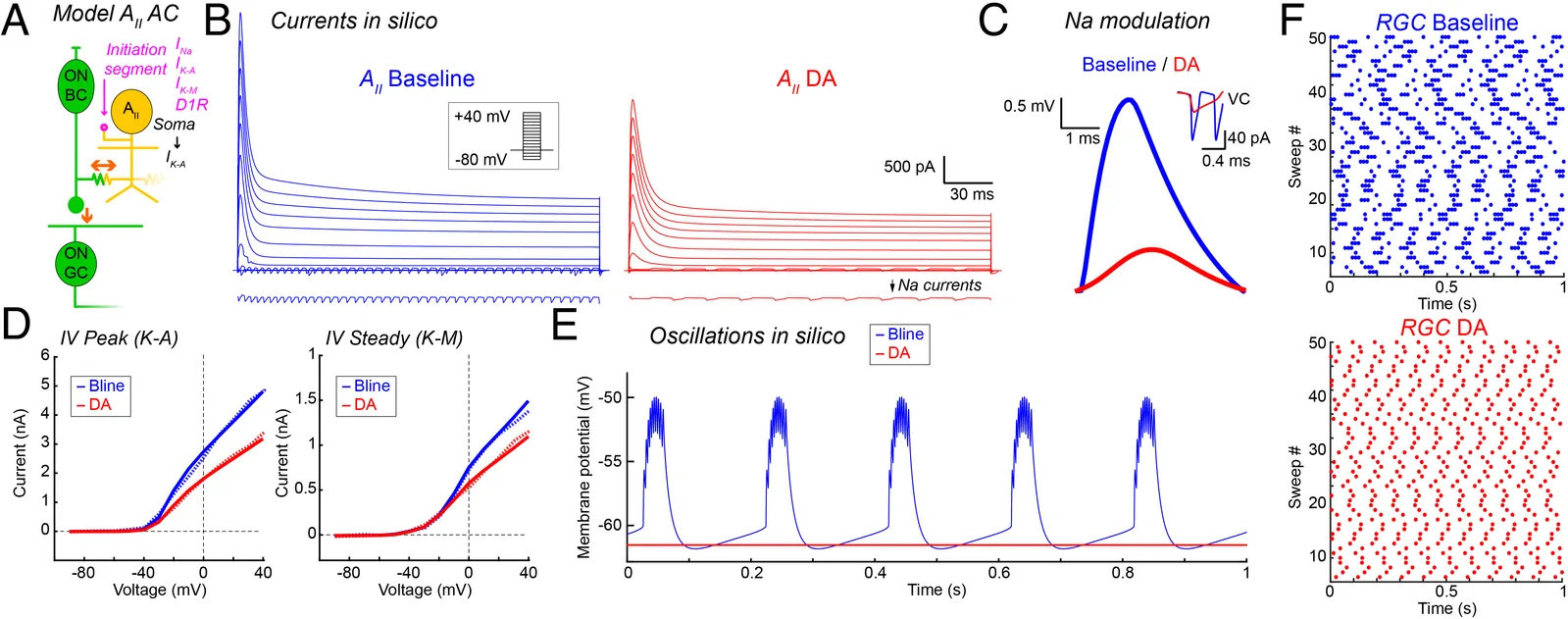
Computational model of a retinal microcircuit recapitulates dopaminergic modulation of excitability and oscillations. (*A*) Schematic of the model consisted of an ON bipolar cell (ON BC), an A_II_ amacrine cell (A_II_ AC), and an ON ganglion cell (ON GC), showing voltage-gated sodium current (I_Na_), A-type potassium current (I_K−A_), M-type potassium current (I_K−M_), and the D1 receptor (D1R) at the initial segment, and a somatic I_K−A_. (*B*) Simulated A_II_ AC voltage-clamp responses to depolarizing voltage steps (−80 mV to +40 mV). *Left*: Baseline (blue traces). *Right*: Simulated effect of dopamine (DA model) application (red). *Bottom* traces show isolated responses to a −50 mV step, highlighting simulated I_Na_-mediated spikelets and their reduction by DA. (*C*) Simulated sodium-dependent activity. Main: Isolated spikelet recorded in current-clamp for baseline (blue) and DA (red) conditions, demonstrating reduced amplitude and slowed kinetics. *Inset*: Corresponding simulated current modulation (I_Na_) in voltage-clamp. (*D*) Model I–V relationships. *Left*: Peak inward (I_Na_) and outward (I_K−A_) currents. *Right*: Steady-state outward current (I_K−M_). Solid lines are model simulations, and dashed lines are experimental data from a single A_II_ AC for comparison. (*E*) Simulated membrane potential oscillations. Under baseline conditions (blue), the model exhibits robust rhythmic activity within the 5 Hz regime. In the DA condition (red), these oscillations are completely suppressed, stabilizing the AII AC. (*F*) *Top*: raster plot of the model RGC in baseline condition oscillating at approximately 5 Hz. *Bottom*: same RGC in DA condition without oscillatory burst activity.

The model we developed for *Nyx^nob^* A_II_ ACs reproduced both the voltage step responses and the IVs of the transient and sustained currents under baseline conditions (Fig. 3 *A*–*D*). Moreover, reducing the conductance, increasing the inactivation time constant, and reducing the activation/inactivation ratio of the I_K-A_ were sufficient to simulate the DA condition (Fig. 3*B* and Table 1). To account for the changes in shape of the spikelets under current clamp conditions, additional changes in I_Na_ were needed. The spikelets are generated by the combined activity of I_Na_, I_K-A_, and I_K-M_. Since I_Na_ and I_K-M_ are located in the initial segment, we do not have reliable direct measurements of these currents, and we had to estimate the related parameters (Fig. 3*D*; see also Fig. 2 *C* and *D*). To reproduce the spikelet response in the DA condition, it was necessary to reduce the conductance and increase both the activation and inactivation time constants of I_Na_ (Table 1).

**Table 1.** A_II_ AC model parameters

A_II_ AC	Control	Dopamine
V_1/2 h Na_	−54.5 mV	−54.5 mV
V_1/2 m Na_	−48 mV	−48 mV
k_h Na_	4 mV	4 mV
k_m Na_	5 mV	5 mV
τ_m Na_	0.01 ms	0.1 ms
τ_h Na_	0.5 ms	4 ms
E_Na Hand_	50 mV	50 mV
E_K AII_	−68 mV	−68 mV
V_1/2 m K_	−20 mV	−20 mV
k_m K_	4 mV	4 mV
τ_m k_	50 ms	50 ms
V_1/2 hA K_	−62 mV	−62 mV
V_1/2 mA K_	−38 mV	−38 mV
V_1/2 c_	150 mV	150 mV
τ_h1A_	50 ms	50 ms
τ_h2A_	2 ms	4 ms
k_hA K_	6 mV	6 mV
k_mA K_	5 mV	6 mV
k_c_	45 mV	45 mV
τ_mA_	0.7 ms	0.7 ms
f_0_	0.96	0.9
G_K A soma_	11.8 pS	5.88 pS
G_leak Soma_	12.3 pS	12.3 pS
C_Soma_	1.96 nF	1.96 nF
G_Na hand_	3.02 nS	0.50 nS
G_K M hand_	452 pS	452 pS
G_K A hand_	1.51 nS	503 pS
G_leak Hand_	78.5 pS	78.5 pS
E_leak_	− 40 mV	−40 mV
C_Hand_	12.6 pF	12.6 pF
R_1_	4.24 GΩ	4.24 GΩ
R_2_	4.25 GΩ	4.25 GΩ
G_leak Arm_	188 pS	188 pS
C_Arm_	30.2 pF	30.2 pF

Next, we investigated how the oscillatory behavior of the model A_II_ ACs is affected by the DA-related changes in model parameters. The computational model shows that modulation of the ionic currents has, as described above, a pronounced effect on the oscillatory behavior of the A_II_ ACs (Fig. 3*E*). Whereas the baseline model exhibited robust oscillations that increased in frequency (e.g., from ~5 to ~9 Hz) with increasing current injection, the DA-modulated model remained stable over almost the whole current injection range. Only with the strongest current injection, some bursting activity occurred.

Critically, the model also predicts the effect on retinal output. As shown by the modeled RGC activity (Fig. 3*F*), the baseline condition shows oscillatory activity approximately at 5 Hz. These oscillations disappear when the model parameters are switched to the DA-condition (Fig. 3 *F*, *Bottom*). These simulations show that the DA-induced changes in A_II_ AC activity we observed experimentally are necessary and sufficient to account for the DA-dependent abolishment of the pathological oscillatory behavior in *Nyx^nob^* mice.

To understand which feature of which current governed the oscillation frequency, we performed a parameter sweep spanning the baseline and DA conditions (*SI Appendix*, Fig. S4). *SI Appendix*, Fig. S4 shows that the most important change is found in the kinetics of the currents when going from baseline to dopamine. Since the membrane potential hardly changes, this indicates that the burst frequency is mostly governed by this slowing down of the currents. A second observation is that I_K-A_ in the soma shows an overshoot, which coincides with the slow depolarizing phase in the oscillations (for an in-depth analysis, see supplementary data; *SI Appendix*, Fig. S4*E*).

### DA Enhances RGC Light Response Fidelity.

**Fig. 4.**
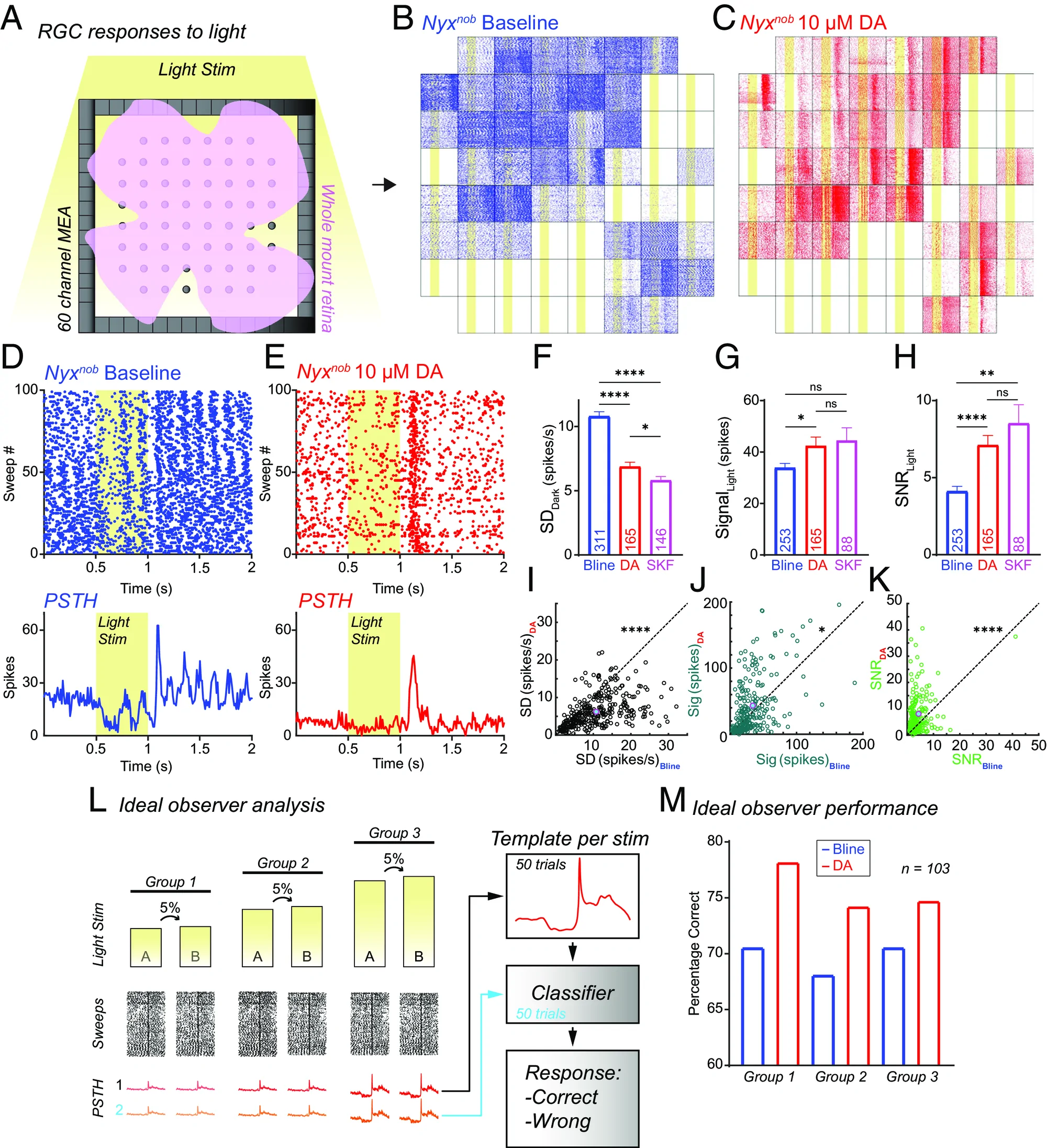
Dopamine enhances RGCs light response fidelity in *Nyx^nob^* mice. (*A*) Schematic of retina MEA recording with a 500 ms light stimulation, repeated every 2 seconds. (*B* and *C*) Global RGC activity raster plots. (*B*) Baseline *Nyx^nob^* retina (blue), with oscillatory background activity and modest light responses. (*C*) Same *Nyx^nob^* retina after 10 µM dopamine (DA, red), showing suppressed background oscillations and enhanced light responses. (*D*) Single RGC’s response to light stimulation (baseline). *Top*: Raster plot (blue). *Bottom*: Corresponding peristimulus time histogram (PSTH). (*E*) Same RGC as in (*D*) after 10 µM DA (red), showing reduced baseline activity and increased peak light response. (*F*) Mean SD of baseline RGC firing rates (noise) under baseline (blue), 10 µM DA (red), and 10 µM SKF-81297 (SKF; magenta). Both DA and SKF significantly reduce baseline noise (*P* < 0.0001, Wilcoxon test), with a significant difference between DA and SKF (*P* = 0.0457, Mann–Whitney test; see text for values). (*G*) Mean peak light response signal under baseline (blue), 10 µM DA (red), and 10 µM SKF (magenta). DA significantly increased signal strength (*P* = 0.038), whereas SKF did not (*P* = 0.470, Wilcoxon test). (*H*) Mean signal-to-noise ratio (SNR) for baseline (blue), 10 µM DA (red), and 10 µM SKF (magenta). Both DA and SKF significantly increase SNR (*P* < 0.0001 for DA, *P* = 0.0023 for SKF, Wilcoxon test), with no significant difference between DA and SKF (*P* = 0.7712, Mann–Whitney test; see text for values). (*I*–*K*) Scatter plots of paired comparisons for individual RGCs. (*I*) Baseline SD after DA versus baseline (SD_DA_ vs. SD_Bline_; black open circles). (*J*) Peak light response signal after DA versus baseline (Sig_DA_ vs. Sig_Bline_; teal open circles). (*K*) SNR after DA versus baseline (SNR_DA_ vs. SNR_Bline_; green open circles). In all scatter plots (*I*–*K*), the cyan-filled purple circle represents the mean of the x and y values. All summary data in bar graphs are mean ± SEM. (*L*) Schematic of ideal observer analysis. Masks are generated from the PSTH of half the trials (50 trials). A classifier then matches the PSTH of the other half of the responses to the masks (Correct/Wrong). This measures discriminability across three intensity groups. (*M*) Percentage Correct classification under baseline (Bline, blue) and 10 µM DA (DA, red). DA consistently increases correct classifications across all groups, demonstrating enhanced RGC discrimination (n = 103 RGCs).

The spontaneous oscillations in *Nyx^nob^* RGCs could potentially interfere with the encoding of visual information by increasing the “noise”. To investigate this, we compared RGC responses to a 500 ms light flash in baseline conditions and in the presence of 10 µM DA or SKF-81297 (Fig. 4). In baseline *Nyx^nob^* retinas, the RGC activity in the dark was strongly oscillatory, whereas the light responses of the RGCs were often very small (Fig. 4 *B* and *D*). However, upon application of DA, the background oscillations were suppressed, and in addition, in some cases, the light responses of the RGCs were significantly larger (Fig. 4 *C* and *E*). To further quantify this effect, we first measured the SD of the baseline firing rate of the RGC activity during a 5 s long dark period preceding the light flash. All individual traces were aligned such that the oscillations were in phase. We consider the SD identified this way as a measure of the noise. Fig. 4 *F* and *I* show that the SDs after DA or SKF-81297 applications were significantly reduced (*P* < 0.0001 for both SD_Bline_ = 10.8 vs. SD_DA_ = 6.9 and SD_Bline_ vs. SD_SKF_ = 5.8, Wilcoxon test), with a significant difference between DA and SKF-81297 (*P* = 0.046, Mann–Whitney test). Instead, application of the D2-agonist QPH had no effect on the SD of the baseline firing (*SI Appendix*, Fig. S2*F*).

Next, we determined the amplitude of the peak offset light-response for all RGCs without further classification. This is a crude measure for the amplitude of the light-response, further referred to as the signal. DA significantly increased this signal strength, whereas the D1-agonist SKF-81297 did not show a statistically significant effect (Fig. 4*G*; *P* = 0.038 for Bline vs. DA, *P* = 0.470 for Bline vs. SKF, *P* = 0.976 for DA vs. SKF). Fig. 4*J* shows the paired comparison of the signal between control and DA (baseline: 33.93 spikes vs. DA: 43.18 spikes, n = 253 pairs, *P* = 0.038, Wilcoxon test). We calculated the SNR by dividing the signal by the SD of the dark activity for each recorded RGC. Both DA and SKF-81297 significantly increased the SNR compared to the baseline condition (Fig. 4 *H* and *K*, *P* < 0.0001 for DA, *P* = 0.002 for SKF, SNR_Bline_ = 4.1, SNR_DA_ = 7.1, SNR_SKF_ = 8.5, Wilcoxon test). To quantify the relative contributions of these changes, we calculated the natural logarithm of the fold changes. Consistent with the paired comparisons shown in the scatter plots (Fig. 4 *I*–*K*), we pooled the DA and SKF-81297 data for this analysis. Across this combined dataset, the treatment resulted in a relative reduction in noise [ln(SD_DA_/SD_Bline_) = −0.53; SEM = 0.05] and a relative increase in signal amplitude [ln(Sig_DA_/Sig_Bline_) = 0.24; SEM = 0.07]. Because our earlier analysis demonstrated that SKF-81297 alone does not significantly increase signal strength, this confirms that removing the oscillations is the primary D1-mediated mechanism for the enhanced retinal output fidelity. Interestingly, application of the D2-agonist QPH also led to an increase in the SNR (*SI Appendix*, Fig. S2 *G*, *P* < 0.0001 for QPH, *SNR_Bline_* = 5.4, *SNR_QPH_* = 9.4, Wilcoxon test). Since the baseline noise was not significantly affected by QPH, this improvement in SNR must be attributed to an increase in the signal. The origin of the increased signal by D2 DA agonists is most likely not due to modulation of the A_II_ AC, since D2 receptors have not been found on these neurons (22). Yet, D2 receptors are abundantly present in the outer and inner plexiform layer, giving ample opportunity for modulation of the upstream signals for the RGCs (22).

So far, the analysis showed how the SNR changed in DA conditions. To link this result to visual performance, we used an ideal observer approach and determined how DA affected the discriminative power of RGCs (Fig. 4*L*). Hundred responses to two flash stimuli of almost equal intensity (5% difference) were recorded on the MEA before and after application of DA. This dataset was used to train and test the ideal observer. Specifically, fifty responses (odd-numbered trials) were used to generate a template PSTH for each recorded unit, and the remaining fifty responses (even-numbered trials) were used as a dataset for the classifier. For each test response, the sum of squared errors between the templates for stim A and B and the dataset was calculated. The lowest sum of squared errors was used to classify the response to be most likely generated by stimulus A or B. In this way, we could generate a histogram for the detection of either stimulus A or B. Fig. 4*M* shows that application of DA markedly increases the discriminative power of RGCs. Thus, our data show that DA application in *Nyx^nob^* mice does not only suppress the retinal oscillations but also improve the signal quality of RGCs by lowering the noise via D1 receptors and increasing the signal via both D1 and D2 receptors.

## Discussion

**Fig. 5.**
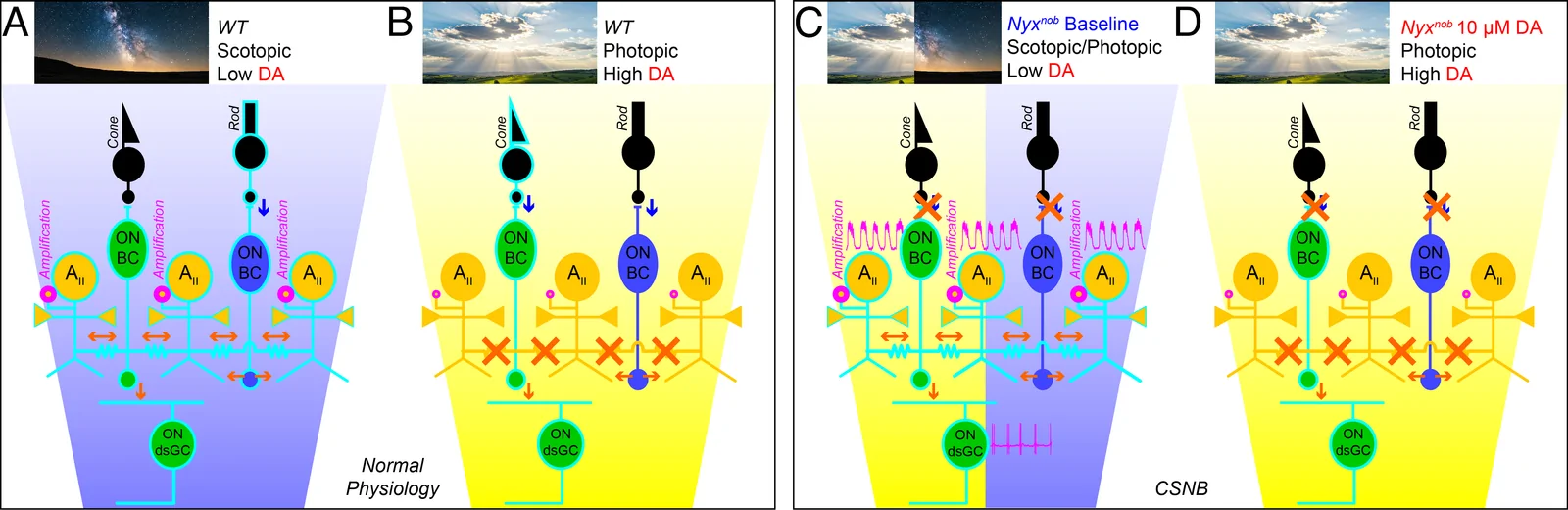
Schematic summary of dopaminergic control of A_II_ amacrine cell activity and its implications for nystagmus in CSNB. The A_II_ amacrine cell (AC) microcircuit depicts a cone and a rod providing input to their respective ON bipolar cells (ON-BCs). These ON-BCs connect to A_II_ ACs, interconnected by gap junctions and provide input to ON retinal ganglion cells (ON-RGCs) including the ON directional RGCs (ON-dsRGCs). Signal flow is indicated by arrows: blue are sign-inverting, and red are sign-conserving pathways. Active cells and pathways are highlighted. (*A*) Scotopic (Low DA): rods transmit light signals, relayed via rod ON-BCs to A_II_ ACs. A_II_ ACs are electrically coupled to each other and cone ON-BCs, and their initial segments are highly sensitive, amplifying weak rod signals for transmission to RGCs. (*B*) Photopic (High DA): Dopamine levels are high; cones transmit light signals, activating cone ON-BCs. High DA levels reduce the coupling between A_II_ ACs and desensitize their initial segments, preventing unnecessary amplification of robust cone signals and ensuring stable retinal output. (*C*) Photopic or scotopic conditions in CSNB (*Nyx^nob^*): The genetic defect disrupts the photoreceptor to ON-BC synapse, leading to a dysfunctional ON-pathway and pathologically low retinal DA levels. Consequently, the retina remains in its scotopic wiring even in the photopic condition. A_II_ ACs remain coupled, and their initial segments remain hypersensitive. This renders A_II_ ACs unstable, causing them to generate spontaneous oscillations which are transmitted to ON-dsRGCs, ultimately driving nystagmus. (*D*) Pharmacological intervention in CSNB (*Nyx^nob^*) with DA: Application of DA increases retinal DA levels shifting the retina to its photopic wiring. This exogenous DA reduces the sensitivity of the A_II_ AC initial segments and likely decreases their coupling. As a result, A_II_ ACs are stabilized; their spontaneous oscillations cease.

In this paper, we show that activation of D1 DA receptors in the retina of *Nyx^nob^* mice reduces the sensitivity of A_II_ ACs by affecting their I_KA_ and I_Na_ channels and thereby their propensity to oscillate (Fig. 5). As a consequence, DA modulation effectively blocks oscillations of the A_II_ ACs, preventing oscillatory activity from propagating to the RGCs and from there to the AOS. The block of the oscillations led to a significant increase in SNR of the RGCs and increased the discriminative power of RGCs signals.

DA is known to modulate the gap junctions among A_II_ ACs and between A_II_ ACs and ON-BCs (22). Could this modulation account for our results? Let us consider two cells (cell 1 and cell 2) coupled by a gap junction R(_gap_) with each cell having a particular membrane resistance (R_m_) and capacitance (C_m_). If all current is injected into cell 1 while the gap junction is fully closed (R_gap_ = inf), only C_m_ of cell 1 will change and the responses will be big and fast. Instead, if R_gap_ has a finite value, part of the current injected into cell 1 will leak into cell 2 via R_gap_, leading to a reduction of the current flowing into R_m_ and charging C_m_ of cell 1. The consequence is that the response will be smaller and slower. So, if the effects we see are induced by dopamine closing the gap junction, we would have expected an increase in time constants and amplitudes. We see the opposite.

Furthermore, DA did not affect the resting membrane potential, which excludes the possibility that sustained hyperpolarization inhibited the oscillatory behavior of the A_II_ ACs. This leaves the modulation of the voltage-gated ion channels as the likely source of the change in oscillatory behavior. Indeed, DA can, depending on the concentration, increase (low concentration) or reduce (high concentration) the max conductance of potassium channels (32). We found a reduction in the peak current and a slowing down of I_K-A_, a modulation not found by Veruki and colleagues in the rat retina (18). With regard to modulation of the I_Na_ channel, we found that the maximum conductance can be reduced by D1 DA receptor activation, which is similar to what Veruki and colleagues found in A_II_ ACs in rat retina (18) and what others observed in hippocampal pyramidal neurons (33). Likewise, we found that the spikelets are attenuated in amplitude and pace by DA, which is in line with findings by Hayashida and colleagues in RGCs of rats (34).

Our model simulations indicate that the changes we observe in the voltage-gated channels in the A_II_ ACs are necessary and sufficient to account for the change in oscillatory behavior of the A_II_ ACs and thus for the modulation of the RGC oscillations and nystagmus. Still, DA can in principle also affect other processes and cells in the retina (22), and we can therefore not exclude the possibility that the light-response properties of the RGCs are also affected by dopaminergic modulation of one or more of the other retinal neurons. Our results support the hypothesis that dopamine acting via a D1 receptor on A_II_ ACs changes the oscillatory behavior of these neurons, while the signal strength seems to be affected by activation of D2 receptors on other retinal neurons, including RGCs.

For the current study, we used a mouse model of CSNB. So, what is the link between CSNB and dopamine? CSNB mice suffer from low levels of DA in their retina due to defects in their rod ON-pathway, which is known to regulate DA release (27–30). Based on our results, we propose that this loss of DA input to the A_II_ ACs overly sensitizes their voltage-gated ion channels in the light-adapted condition, making these neurons prone to spontaneous oscillations (Fig. 5*C*). The retinas of CSNB mice have lost their primary rod pathway, which leads to a reduced DA release (28), putting their retinas into a chronically dark-adapted state. This makes the retina highly unstable under photopic conditions. Since the retinal oscillations eventually cause nystagmus, our findings provide a cellular and molecular mechanism linking the retinal defect in CSNB to the manifestation of nystagmus.

CSNB patients often suffer from poor visual performance (35). Our results suggest a possible cause for this. Suppressing the spontaneous oscillations markedly improved the SNR of the RGC light responses in *Nyx^nob^* mice, which most likely has direct implications for visual performance. While developmental factors such as desegregation of retino-thalamic axons in the dorsolateral geniculate nucleus may affect the visual performance (36), our results suggest that low SNR at the retinal level, caused by the persistent background oscillations and low signal strength, also forms a significant contributing factor. By reducing the oscillations (noise) and enhancing the amplitude of the light responses (signal), DA application not only blocks the oscillatory eye movements, but also increases the SNR of the retinal output, offering a dual benefit for both stabilizing gaze and improving visual function.

Our finding that oscillating A_II_ ACs are the main driver of nystagmus associated with CSNB (11, 13) raises the question as to whether A_II_ AC instability could be a common underlying mechanism for nystagmus in a broader range of syndromes. A_II_ ACs are neurons that relay rod signals to the RGCs. A delicate balance between the activity of the voltage-gated ion channels on the initial segment, the DA release, and the membrane potential of the A_II_ ACs is needed to keep the A_II_ ACs stable. In many diseases, this balance may be disturbed. A_II_ ACs are highly interconnected (Fig. 5 and *SI Appendix*, Fig. S1). Their main input is a glutamatergic input from ON rod-BCs, but they also receive inhibitory and neuromodulatory inputs, and they are also electrically coupled with other A_II_ ACs and cone ON-BCs. We hypothesize that any substantial depolarization of the A_II_ AC resting membrane potential due to a change in their synaptic input or disturbance in the dopaminergic input of the A_II_ ACs will lead to oscillations of A_II_ ACs and downstream RGCs, and thereby induce nystagmus.

In the rd1 mouse model, A_II_ ACs oscillate with a frequency of about 10 Hz (21). In these mice, photoreceptors, especially rods, have degenerated. The oscillations are considered to be generated by disturbance of the input to the A_II_ ACs because of the lack of functional photoreceptors. However, it is highly likely that the dopaminergic system is also affected by the lack of photoreceptor input, thus contributing to the oscillations.

There are several reports indicating nystagmus in retinitis pigmentosa (37–39), but not all retinitis pigmentosa patients have nystagmus. What is the origin of this variability? We propose that three conditions have to be met for nystagmus to occur: 1) oscillating A_II_ ACs and RGCs, 2) oscillations should occur over a substantial area of the retina, and 3) synchronized oscillations of RGCs. It could be that during the progression of retinitis pigmentosa, the time window where A_II_ ACs and RGCs oscillations occur over a substantial area of the retina and the time window when enough photoreceptors are still functional to synchronize the oscillations only overlap for relatively short and variable periods.

Several other syndromes present with nystagmus or oscillating eye movements and involve disruptions that could impact the delicate A_II_ AC balance. In albinism, the retinal dopaminergic system may be disturbed (40), which may have reduced the dopaminergic inputs to the A_II_ ACs and upregulated the voltage-gated ion channels, which could lead to oscillations and nystagmus. Similarly, the oscillating eye movements often found in Parkinson’s disease (41) may be directly due to reduced dopaminergic inputs to the A_II_ ACs. In early-onset severe retinal dystrophies, nystagmus could arise from disturbed glutamatergic inputs to the ON-BCs leading to depolarization of the A_II_ ACs (21). Moreover, observations in a mouse model of multiple sclerosis, indicating reduced photoreceptor synaptic output (42–44), also point toward aberrations in A_II_ AC depolarization, oscillations, and nystagmus.

Could our findings also offer insight into the cause of myopia, a common symptom in CSNB patients? Goethals and colleagues (45) have proposed that local contrast detector neurons in the retina may signal signs of defocus and thereby regulate growth of the eyes. Other mechanisms to detect defocus, such as longitudinal chromatic aberration and monochromatic aberration, have been suggested as well (46). Yet, independent of the mechanism that detects defocus, activity in the neurons performing this task has been consistently proposed to stimulate eye growth. In the retina, many ACs, BCs, and RGCs are connected to A_II_ ACs, and all these connected neurons receive an oscillatory input. It is therefore tempting to consider the possibility that in CSNB the oscillatory activity in the defocus-detecting neurons may promote eye growth, leading to myopia, akin to the impact of flickering light on axial elongation of eyes in WT mice (47–49).

The results presented in this paper could potentially lead to the first noninvasive, pharmacological treatment targeting infantile nystagmus associated with CSNB. Given that activation of D1 receptors blocks the A_II_ ACs and RGC oscillations and increases the RGC signal fidelity, application of D1 agonists or a combination of D1 and D2 agonists via eye drops might form a potential treatment of nystagmus. Even though such treatment will not cure CSNB, it may dramatically reduce the burden of some of the symptoms, such as nystagmus, low visual performance, and possibly even myopia. The absence of rod vision will remain, but vision at the cone threshold may improve, because of the increased SNR of the RGC signals. While validation in other infantile nystagmus models and long-term efficacy studies are necessary, this work provides significant mechanistic understanding into infantile nystagmus pathophysiology and presents topical dopaminergic activation—specifically D1 agonists—as a promising and testable therapeutic strategy, warranting further preclinical investigation.

## Materials and Methods

Many procedures used in this study were described previously in Winkelman et al. (13) and Holzel et al. (11).

### Animals.

Animal experiments were approved by the ethical committee of the Royal Netherlands Academy of Arts and Sciences (KNAW) in accordance with the European Communities Council Directive (2010/63/EU) under licenses AVD-801002016517 and AVD-80100202115698 (Central Committee for Animal Experiments of the Netherlands).

*Nyx^nob^* mice were obtained from the McCall lab (University of Louisville, Louisville). *Nyx^nob^* and WT mice were in a C57BL/6JRj background. Since the Nyx mutation is x-linked, only male mice in the age range of 5 to 71 wk were used for the experiments. Room lights were timed on a 12/12 h light–dark schedule, and experiments took place during daytime. Mice had ad libitum access to food and water.

### Multielectrode RGC Recordings.

#### Retinal dissection.

After 1 h of dark adaptation, mice were sedated with CO_2_/O_2_ and euthanized by cervical dislocation. All procedures were performed under dim red light. Eyes were enucleated and placed in room-temperature Ames’ medium (Sigma-Aldrich, St Louis, MO). The cornea and lens were removed by making an incision around the *ora serrata* using fine spring scissors. As much vitreous humor as possible, as well as the sclera was removed using fine forceps. Four small incisions were made, and the retina was flat-mounted on a filter paper annulus (3 mm inner radius; 0.8 µm hydrophilic MCE MF-MilliporeTM membrane filter, Merck Millipore Ltd., Tullagreen, Ireland). The retina was then mounted photoreceptor cell side up on a perforated 60-electrode MEA chip (60pMEA200/30iR-Ti, Multichannel systems, Reutlingen, Germany) in a recording chamber mounted on a Nikon Optiphot-2 upright microscope and viewed under IR with an Olympus 2x objective and video camera (Abus TVCC 20530). Retinas were continuously superfused with Ames’ medium gassed with a mixture of O_2_ and CO_2_ at a pH of 7.4 and a temperature of 35 to 37 °C at a flow rate of 1.5 mL/min. Before the recording started, an acclimatization period in the dark for a duration of 15 min was given to ensure stable recordings.

#### Data acquisition.

The extracellular RGC activity (n = 536 units, N = 10 retinas/mice) was acquired using MC rack (Multichannel systems, Reutlingen, Germany) at a sampling frequency of 25 kHz. The data were then zero-phase bandpass filtered (250 to 6250 Hz) with a fourth-order Butterworth filter in Matlab (Mathworks, Natick, MA). Subsequently, the spiking activity was sorted manually into single-unit activity using the Plexon offline sorter (Plexon, Dallas, TX) based on the first two principal components versus time. For the extraction of spikes from the background, a spike detection amplitude threshold of >4σ_n_ was used as a criterion. Here, σ_n_ is defined as an estimation of the SD of background noise and x is the bandpass-filtered signal (11).

$$\sigma_{n}=median\left(\frac{\left|x\right|}{0.6745}\right).$$

#### Optical stimulator.

Full-field white light flashes were generated using Psychophysics Toolbox Version 3 (Brainard, 1997) and presented to the photoreceptor side of the retina with a custom-modified DLP projector [custom modification (50); Light Crafter 4500, Wintech, Carlsbad, CA]. Only white light stimuli were used. White light stimuli consisted of equal quantal output of the red (625 nm), green (530 nm), blue (455 nm), and UV (385 nm) LEDs. The maximal light intensity was 8.60 × 10^16^ quanta m^−2^ s^−1^.

#### Data analysis.

To quantify the oscillatory behavior of RGC’s activity, the firing activity in the dark was recorded for a period of at least 15 min, cut into 5 s nonoverlapping epochs. The software procedure setup described above produces a series of spike times. These datasets were converted into time series with 1 ms resolution. Each epoch was detrended and baseline subtracted, after which the autocorrelation and Welch’s PSD were calculated for each cell per trace and then averaged per cell.

For the signal-to-noise analysis, PSTHs were made from each cell’s activity in 10 ms bins. The SNR was defined as the response divided by the STD. The response was defined as the absolute deviation from the mean after light offset, as we are only analyzing the OFF response due to the absence of an ON response in the *Nyx^nob^* mice. The mean was taken in the 500 ms dark period before the stimulus. The noise was calculated from a 5 s long dark measurement taken before the stimulus protocol. The oscillatory activity in the individual traces was synchronized to the first burst. After this synchronization, a PSTH was made and the STD was calculated. The various traces were aligned such that the bursting activity was synchronized. This was done to obtain a fair measure of the variance in the signal. Summing unsynchronized oscillatory activity will be turned into a small stable signal (as seen in Fig. 4*D*) and would therefore underestimate the noise induced by the oscillations. The synchronized activity seen in the PSTH still dampens out over time due to small variations in the frequency. As a control, we did run the analysis on the unsynchronized data as well and still found an increase in the SNR although smaller.

Time constants were determined by fitting a double exponential curve through the voltage clamp data, using the following equation.

$$y=c+A_{1}e^{\frac{-x}{\tau_{1}}}+A_{2}e^{\frac{-x}{\tau_{2}}}.$$

### Ideal Observer.

We investigated the effect of dopamine on the signal quality with an ideal observer type of analysis. For this analysis, we stimulated the retina with 3 groups of 2 stimuli that differed in intensity by 5%. The intensity of the groups differed by 0.33 log units. These stimuli were repeated 100 times. The data were split into 2 datasets consisting of the even and the odd numbered traces from which PSTHs were made. The even traces were used as data, and the odd numbered traces to generate masks of the responses per cell. The masks were created by fitting straight lines through segments of the dataset. We chose the segments based on the steady and dynamic parts of the response. The steady parts, 0 to 550 ms, 1,500 to 1,600 ms, and 1,600 to 2,000 ms, were fitted with single straight lines. The dynamic parts, 550 to 650 and 1050 to 1500, were fitted with a line between every 3 data points. Finally, we took the sum of the squared errors between the response and the mask to determine which mask fits the response best and used that as a quantity representing the ability to discriminate between the 2 stimuli.

### Statistical Analysis.

Statistical analyses were performed using GraphPad Prism 10.0.3 (GraphPad Software, Massachusetts) or Matlab (MathWorks Inc., Natick, MA). All mean values are presented ± SEM. Statistical significance was tested using a Wilcoxon matched-paired signed-rank test for paired data and a Mann–Whitney test for unpaired data. Normality was tested using the Shapiro–Wilk test.

Outliers were defined as values deviating more than 3 SD from the mean. The outliers were replaced by the mean of the population.

### Whole Cell A_II_ AC Recordings.

For recordings of A_II_ ACs (n = 19 cells, N = 15 mice), vertical slices of mouse retina (~200 µm) were prepared using a custom-made tissue slicer. Animals were dark-adapted for 1 h. Anesthetization and all subsequent procedures were performed under infrared illumination to preserve the adaptation state. During recordings, cells were visualized and targeted under infrared illumination. Slices were transferred to the recording chamber and perfused at a rate of 1.5 mL/min with bicarbonate-buffered Ames medium (Sigma-Aldrich) continuously gassed with a mixture of O_2_ and CO_2_ at a pH of 7.4. Slices were visualized with a Nikon Eclipse E2000FN microscope fitted with a 60x/1.0 water immersion objective with an infrared differential interference contrast, a video camera (Philips, The Netherlands), and a two-photon scan head. A_II_ ACs were identified by their location, morphology, and characteristic current responses. Recordings were performed at 35 to 37 °C. Series resistance, which was always below 30 MΩ, was not compensated, and the liquid junction potential was not corrected.

Patch electrodes were fabricated from thick-walled borosilicate glass to have a resistance of 5 to 7 MΩ. The intracellular solution contained (in mM): K-methanesulfonate, 135; KCl, 6; Na2-ATP, 2; Na-GTP, 1; EGTA, 1; MgCl2, 2; Na-HEPES, 5; and adjusted to pH 7.35 with KOH. Lucifer yellow (1 mg/mL) was added to these solutions for morphological identification of recorded cells using a two-photon microscope. Whole-cell recordings were made with a Multiclamp 700B amplifier using a Digidata 1322A digitizer (MDS Analytical Technologies, Union City, CA) and Signal 6.04 software (Cambridge Electronic Design Ltd, Cambridge, UK) to generate command outputs, trigger the light stimulus, and acquire and analyze analog whole-cell voltages. These data were sampled at 10 kHz and filtered at 2.4 kHz with a four-pole Bessel low-pass filter. Matlab (MathWorks Inc., Natick, MA) and Igor Pro (WaveMetrics, Portland, OR) were used to analyze the data.

### Computational Model.

Fig. 3*A* illustrates the simulated network composed of an ON-BC, A_II_ AC, and RGC. In this network, A_II_ ACs form electrical connections via gap junctions with cone-driven ON-BC (ON-CBC). The A_II_ AC receives excitatory glutamatergic input from ON-RBCs (15) and inhibitory input from OFF-ACs that are activated by OFF-CBCs (15). This inhibitory connection represents a form of crossover inhibition. When light is presented, ON BCs depolarize, which consequently depolarizes the A_II_ ACs. At the same time, light stimulation causes OFF-CBCs and thus the OFF-ACs they drive to hyperpolarize, reducing their inhibitory influence on A_II_ ACs and further enhancing A_II_ AC depolarization. ON-CBCs transmit signals to ON-RGCs, whereas OFF-BCs project to OFF-RGCs. In the model, there is only one ON-CBC per AII AC although literature shows that one A_II_ AC is contacted by 10 ON-CBCs (51). This physiological connectivity was implemented by scaling the coupling conductance. Next, we present the equations describing the various cell types.

#### A_II_ AC model.

The A_II_ AC was modeled using a three-compartment framework adapted from Choi and colleagues (21). This model includes a soma, an initial segment, and an interconnecting cable. The dendritic arbor is represented as part of the somatic compartment. All ionic currents in the model are governed by Hodgkin–Huxley-type equations.

The sodium current (*I_Na_*) is given by

$$I_{\mathrm{Na}}=G_{\mathrm{Na}}m_{\mathrm{Na}}^{3}h_{\mathrm{Na}}(V-E_{\mathrm{Na}}),$$

where the gating variables are

$$\tau_{\mathrm{mNa}}\frac{dm_{\mathrm{Na}}}{\mathrm{dt}}=m_{\infty Na}-m_{\mathrm{Na}},$$

$$\tau_{\mathrm{hNa}}\frac{dm_{\mathrm{Na}}}{\mathrm{dt}}=h_{\infty Na}-h_{\mathrm{Na}},$$

with

$$m_{\infty Na}\left(V\right)={\left(1+{e^{-}}^{\frac{V+V_{\frac{1}{2}}mNa}{5}}\right)}^{-1},$$

$$h_{\infty Na}\left(V\right)={\left(1+{e^{-}}^{\frac{V+V_{\frac{1}{2}}hNa}{2}}\right)}^{-1}.$$

The M-type potassium current (*I_M_*) is given by

$$I_{M}=G_{M}m_{M}(V-E_{K}),$$

where the gating variables are

$$\tau_{\mathrm{mM}}\frac{dm_{M}}{\mathrm{dt}}=m_{\infty M}-m_{M},$$

with

$$m_{\infty Na}\left(V\right)={\left(1+{e^{-}}^{\frac{V+V_{\frac{1}{2}}mK}{4}}\right)}^{-1}.$$

The A-type potassium current (*I_A_*) is given by

$$I_{A}=G_{A}m_{A}\left(ch_{1A}+\left(1-c\right)h_{2A}\right)(V-E_{K}),$$

$$c={\left(1+{e^{-}}^{\frac{V+V_{\frac{1}{2}}c}{k_{c}}}\right)}^{-1},$$

where the gating variables are

$$\tau_{\mathrm{mA}}\frac{dm_{A}}{\mathrm{dt}}=m_{\infty A}-m_{A},$$

$$\tau_{h_{1A}}\frac{dh_{1A}}{\mathrm{dt}}=h_{\infty A}-h_{1A},$$

$$\tau_{h_{2A}}\frac{dh_{2A}}{\mathrm{dt}}=h_{\infty A}-h_{2A},$$

with

$$m_{\infty A}\left(V\right)={\left(1+{e^{-}}^{\frac{V+V_{\frac{1}{2}}mAK}{k_{\mathrm{mAK}}}}\right)}^{-1},$$

$$h_{\infty A}\left(V\right)={\left(1+{e^{-}}^{\frac{V+V_{\frac{1}{2}}hAK}{k_{\mathrm{hAK}}}}\right)}^{-1}+\left(1-f\right),$$

where *f* is defined as

$$f=f_{0}\left(1-\frac{0.07}{1+e^{-\frac{\left(V+20\right)}{5}}}\right).$$

Next to these ionic currents, there is a leak current

$$I_{leak,AII}={\bar{g}}_{\mathrm{leak}}\left(V-E_{\mathrm{leak}}\right).$$

The ON-CBCs are connected to the A_II_ ACs through gap junctions. For computational efficiency, each A_II_ AC was coupled with a single ON-CBC in the simulation. To represent the biological condition in which one A_II_ AC is coupled to approximately 10 ON-CBCs, the gap junction conductance on the A_II_ AC side was set to be 10 times greater than that experienced by the ON-CBC.

The gap junction currents are given by

$$I_{\mathrm{GJ}}=G_{\mathrm{GJ}}\left(V_{\mathrm{Coupled}}-V\right).$$

The membrane potentials of the three compartments

were calculated as

$$C\frac{dV_{\mathrm{IS}}}{\mathrm{dt}}=-\left(I_{\mathrm{Na}}+I_{A}+I_{M}+I_{\mathrm{leak}}\right)+I_{\mathrm{compart}},$$

$$C\frac{dV_{\mathrm{Soma}}}{\mathrm{dt}}=-\left(I_{A}+I_{\mathrm{leak}}\right)+I_{\mathrm{compart}}+I_{\mathrm{GJ}}+I_{\mathrm{inject}},$$

$$C\frac{dV_{\mathrm{cable}}}{\mathrm{dt}}=-I_{\mathrm{leak}}+I_{\mathrm{compart}}.$$

The model contains too many parameters for an automated fitting procedure due to the presence of multiple local minima. Furthermore, the voltage clamp data are not exact due to a space clamp problem between the soma and the initial segment. One of the consequences of this is that the voltage clamp data contain unclamped spikelets generated in the initial segment. Therefore, we manually fitted the model to the data. An additional benefit of such a procedure is that it generates understanding of the model behavior and therefore can avoid local minima better. First the model was fitted to voltage step responses in a voltage clamp and accompanying IV’s while taking the space clamp problem into account. Without changing the parameters, the current clamp simulations were run and compared to the data. When the later model responses also corresponded to the real data, the parameters were finalized. The model parameters are summarized in Table 1.

#### ON-CBC model.

For the ON-CBC, we used the model from Usui and colleagues (52). This model consists of a delayed rectifying potassium current (*I_Kv_*), a transient potassium current (*I_A_*), a hyperpolarization-activated current (*I_h_*), a calcium current (*I_Ca_*), and a calcium-dependent potassium current (*I_KCa_*). A two-compartment model was used for the internal calcium concentration of the ON-CBC, which is needed to calculate I_KCa_. One compartment was close to the membrane and consisted of a fast and a slow calcium buffer and two exchange currents. The other compartment was within the cell and consisted of only the two calcium buffers. Calcium will flow from one compartment into the other by diffusion.

All ionic currents are described with Hodgkin–Huxley-type equations, except for I_h_ which is described with a Markov chain-type model.

The delayed rectifying potassium current (*I_Kv_*) is described by

$$I_{\mathrm{Kv}}=G_{\mathrm{Kv}}{m_{\mathrm{Kv}}}^{3}h_{\mathrm{Kv}}\left(V-E_{k}\right),$$

where the gating variables are calculated as

$$\frac{dm_{\mathrm{Kv}}}{\mathrm{dt}}=\alpha_{\mathrm{mKv}}\left(1-m_{\mathrm{Kv}}\right)-\beta_{\mathrm{mKv}}m_{\mathrm{Kv}},$$

$$\frac{dh_{\mathrm{Kv}}}{\mathrm{dt}}=\alpha_{\mathrm{hKv}}\left(1-h_{\mathrm{Kv}}\right)-\beta_{\mathrm{hKv}}h_{\mathrm{Kv}},$$

with

$$\alpha_{\mathrm{mKv}}=\frac{400}{1+e^{-\frac{V-15}{36}}},$$

$$\beta_{\mathrm{mKv}}=e^{-\frac{V}{13}},$$

$$\alpha_{\mathrm{hKv}}=0.3\cdot 10^{-3}\cdot e^{-\frac{V}{7}},$$

$$\beta_{\mathrm{hKv}}=\frac{80}{1+e^{-\frac{V+115}{15}}}+0.02.$$

The transient potassium current (I_A_) is modeled with Hodgkin–Huxley type equations and is defined as

$$I_{A}=G_{A}m_{A}^{3}h_{A}\left(V-E_{K}\right),$$

with the gating variables

$$\frac{dm_{A}}{\mathrm{dt}}=\alpha_{\mathrm{mA}}\left(1-m_{A}\right)-\beta_{\mathrm{mA}}m_{A},$$

$$\frac{dh_{A}}{\mathrm{dt}}=\alpha_{\mathrm{hA}}\left(1-h_{A}\right)-\beta_{\mathrm{hA}}h_{A},$$

where

$$\alpha_{mA}=\frac{1200}{1+e^{-\frac{V-50}{28}}},$$

$$\beta_{\mathrm{mA}}=6e^{-\frac{V}{10}},$$

$$\alpha_{\mathrm{hA}}=45\cdot 10^{-3}e^{-\frac{V}{13}},$$

$$\beta_{\mathrm{hA}}=\frac{75}{1+e^{-\frac{V+50}{15}}}.$$

The hyperpolarization activated current (*I_h_*) is described with a Markov chain-like model which describes the transition between open and closed states. The current is given by

$$I_{h}=G_{h}m_{h}(V-E_{h}),$$

where the total open probability is the sum of the open states

$$m_{h}=O_{1}+O_{2}+O_{3}.$$

The open and closed states are calculated as

$$\frac{\mathrm{dM}}{\mathrm{dt}}=KM,$$

where

$$M=\left(\begin{array}{c} C_{1}\\ C_{2}\\ O_{1}\\ O_{2}\\ O_{3} \end{array}\right),$$

$$K=\left[\begin{array}{ccccc} -4\alpha_{h} & \beta_{h} & 0 & 0 & 0\\ 4\alpha_{h} & -\left(3\alpha_{h}+\beta_{h}\right) & 2\beta_{h} & 0 & 0\\ 0 & 3\alpha_{h} & -\left(2\alpha_{h}+2\beta_{h}\right) & 3\beta_{h} & 0\\ 0 & 0 & 2\alpha_{h} & -\left(\alpha_{h}+3\beta_{h}\right) & 4\beta_{h}\\ 0 & 0 & 0 & \alpha_{h} & -4\beta_{h} \end{array}\right].$$

With the transition rate constants defined as

$$\alpha_{h}=\frac{3}{1+e^{\frac{V+110}{15}}},$$

$$\beta_{h}=\frac{1.5}{1+e^{-\frac{V+115}{15}}}.$$

The calcium current (*I_Ca_*) is given by

$$I_{\mathrm{Ca}}=G_{\mathrm{Ca}}m_{\mathrm{Ca}}^{4}h_{\mathrm{Ca}}\left(V-E_{\mathrm{Ca}}\right),$$

with a reversal potential depended on the calcium concentration

$$E_{\mathrm{Ca}}=12.9\text{log}\left(\frac{{\left[{\mathrm{Ca}}^{2+}\right]}_{o}}{{\left[{\mathrm{Ca}}^{2+}\right]}_{s}}\right),$$

with the gating variables

$$\frac{dm_{\mathrm{Ca}}}{\mathrm{dt}}=\alpha_{\mathrm{mCa}}\left(1-m_{\mathrm{Ca}}\right)-\beta_{\mathrm{mCa}},$$

where

$$\alpha_{mCa}=\frac{1200\left(120-V\right)}{e^{\frac{120-V}{25}-1}},$$

$$\beta_{\mathrm{mCa}}=\frac{40\cdot 10^{3}}{1+e^{\frac{V+68}{25}}},$$

$$h_{\mathrm{Ca}}=\frac{e^{-\frac{V-50}{11}}}{1+e^{-\frac{V-50}{11}}}.$$

The calcium-dependent potassium current (*I_KCa_*) is given by

$$I_{\mathrm{KCa}}={\bar{g}}_{\mathrm{Kc}}m_{\mathrm{Kc}}^{2}m_{Kc1}\left(V-E_{K}\right),$$

where the gating variables are described as

$$\frac{dm_{\mathrm{Kc}}}{\mathrm{dt}}=\alpha_{\mathrm{mKc}}\left(1-m_{\mathrm{Kc}}\right)-\beta_{\mathrm{mKc}}m_{\mathrm{Kc}},$$

with

$$\alpha_{\mathrm{mKc}}=\frac{100\cdot \left(230-V\right)}{e^{\frac{\left(230-V\right)}{52}}-1},$$

$$\beta_{\mathrm{mKc}}=e^{-\frac{V}{95}},$$

$$m_{Kc1}=\frac{{\left[{\mathrm{Ca}}^{2+}\right]}_{s}}{{\left[{\mathrm{Ca}}^{2+}\right]}_{s}+0.2}.$$

The change in calcium concentration close to the membrane ([Ca^+2^]_s_) is described as

$$\begin{array}{c} \frac{d[{\mathrm{Ca}}^{2+}{]}_{s}}{\mathrm{dt}}=-\frac{I_{\mathrm{Ca}}}{2FV_{s}}-\frac{D_{\mathrm{Ca}}S_{\mathrm{sd}}}{V_{s}d_{\mathrm{sd}}}\left({\left[{\mathrm{Ca}}^{2+}\right]}_{s}-{\left[{\mathrm{Ca}}^{2+}\right]}_{d}\right)\\ \;-\frac{\left(I_{\mathrm{ex}}-I_{ex2}\right)}{2FV_{s}}+\beta_{\mathrm{bl}}{\left[{\mathrm{Ca}}^{2+}\right]}_{\mathrm{bls}}-\alpha_{\mathrm{bl}}{\left[{\mathrm{Ca}}^{2+}\right]}_{s}\\ \;({\left[{\mathrm{Ca}}^{2+}\right]}_{\mathrm{blmax}}-{\left[{\mathrm{Ca}}^{2+}\right]}_{\mathrm{bls}})+\beta_{\mathrm{bh}}{\left[{\mathrm{Ca}}^{2+}\right]}_{\mathrm{bhs}}-\alpha_{\mathrm{bh}}{\left[{\mathrm{Ca}}^{2+}\right]}_{s}\\ \;({\left[{\mathrm{Ca}}^{2+}\right]}_{\mathrm{bhmax}}-{\left[{\mathrm{Ca}}^{2+}\right]}_{\mathrm{bhs}}), \end{array}$$

and the calcium concentration inside the cell ([Ca^2+^]_d_) as

$$\begin{array}{c} \frac{d{\left[{\mathrm{Ca}}^{2+}\right]}_{d}}{\mathrm{dt}}=\frac{D_{\mathrm{Ca}}S_{\mathrm{sd}}}{V_{d}d_{\mathrm{sd}}}\left({\left[{\mathrm{Ca}}^{2+}\right]}_{s}-{\left[{\mathrm{Ca}}^{2+}\right]}_{d}\right)+\beta_{\mathrm{bl}}{\left[{\mathrm{Ca}}^{2+}\right]}_{\mathrm{bld}}\\ \;-\alpha_{\mathrm{bl}}{\left[{\mathrm{Ca}}^{2+}\right]}_{d}({\left[{\mathrm{Ca}}^{2+}\right]}_{\mathrm{blmax}}-{\left[{\mathrm{Ca}}^{2+}\right]}_{\mathrm{bld}})+\beta_{\mathrm{bh}}{\left[{\mathrm{Ca}}^{2+}\right]}_{\mathrm{bhd}}-\alpha_{\mathrm{bh}}{\left[{\mathrm{Ca}}^{2+}\right]}_{d}\\ \;({\left[{\mathrm{Ca}}^{2+}\right]}_{\mathrm{bhmax}}-{\left[{\mathrm{Ca}}^{2+}\right]}_{\mathrm{bhd}}), \end{array}$$

where

$$\frac{d{\left[{\mathrm{Ca}}^{2+}\right]}_{\mathrm{bls}}}{\mathrm{dt}}=\alpha_{\mathrm{bl}}{\left[{\mathrm{Ca}}^{2+}\right]}_{s}\left({\left[{\mathrm{Ca}}^{2+}\right]}_{\mathrm{blmax}}-{\left[{\mathrm{Ca}}^{2+}\right]}_{\mathrm{bls}}\right)-\beta_{\mathrm{bl}}{\left[{\mathrm{Ca}}^{2+}\right]}_{\mathrm{bls}},$$

$$\frac{d[{\mathrm{Ca}}^{2+}{]}_{\mathrm{bhs}}}{\mathrm{dt}}=\alpha_{\mathrm{bh}}{\left[{\mathrm{Ca}}^{2+}\right]}_{s}\left({\left[{\mathrm{Ca}}^{2+}\right]}_{\mathrm{bhmax}}-{\left[{\mathrm{Ca}}^{2+}\right]}_{\mathrm{bhs}}\right)-\beta_{\mathrm{bh}}{\left[{\mathrm{Ca}}^{2+}\right]}_{\mathrm{bhs}},$$

$$\frac{d[{\mathrm{Ca}}^{2+}{]}_{\mathrm{bld}}}{\mathrm{dt}}=\alpha_{\mathrm{bl}}{\left[{\mathrm{Ca}}^{2+}\right]}_{d}\left({\left[{\mathrm{Ca}}^{2+}\right]}_{\mathrm{blmax}}-{\left[{\mathrm{Ca}}^{2+}\right]}_{\mathrm{bld}}\right)-\beta_{\mathrm{bl}}{\left[{\mathrm{Ca}}^{2+}\right]}_{\mathrm{bld}},$$

$$\frac{d[{\mathrm{Ca}}^{2+}{]}_{\mathrm{bhd}}}{\mathrm{dt}}=\alpha_{\mathrm{bh}}{\left[{\mathrm{Ca}}^{2+}\right]}_{d}\left({\left[{\mathrm{Ca}}^{2+}\right]}_{\mathrm{bhmax}}-{\left[{\mathrm{Ca}}^{2+}\right]}_{\mathrm{bhd}}\right)-\beta_{\mathrm{bh}}{\left[{\mathrm{Ca}}^{2+}\right]}_{\mathrm{bhd}},$$

with the exchange currents defined as

$$I_{\mathrm{ex}}=\frac{J_{\mathrm{ex}}\left({\left[{\mathrm{Ca}}^{2+}\right]}_{s}-{\left[{\mathrm{Ca}}^{2+}\right]}_{\min}\right)}{{\left[{\mathrm{Ca}}^{2+}\right]}_{s}-{\left[{\mathrm{Ca}}^{2+}\right]}_{\min}+2.3}\text{exp}\left(-\frac{V+14}{70}\right),$$

$$I_{ex2}=\frac{J_{ex2}({\left[{\mathrm{Ca}}^{2+}\right]}_{s}-{\left[{\mathrm{Ca}}^{2+}\right]}_{\min})}{{\left[{\mathrm{Ca}}^{2+}\right]}_{s}-{\left[{\mathrm{Ca}}^{2+}\right]}_{\min}+0.5}.$$

Finally, we added a leak current

$$I_{l}=G_{l}\left(V-E_{l}\right).$$

The gap junction between the ON-CBC and the A_II_ AC is given by.

$$I_{\mathrm{GJ}}=G_{\mathrm{GJ}}\left(V_{\mathrm{Coupled}}-V\right).$$

We only simulate one ON-CB per A_II_ cell. The membrane potential of the BC is given by

$$C\frac{dV_{\mathrm{BC}}}{\mathrm{dt}}=-\left(I_{\mathrm{Kv}}+I_{A}+I_{h}+I_{\mathrm{Ca}}+I_{\mathrm{KCa}}+I_{\mathrm{leak}}\right)+I_{\mathrm{GJ}}+I_{\mathrm{inject}}.$$

The model parameters are summarized in Tables 2 and 3.

**Table 2.** ON-CBC and RGC model parameters

ON-CBC	
C	10 pF
*Delayed rectifying potassium current*	
G_Kv_	2 nS
E_Kv_	−58 mV
*The transient potassium current*	
G_A_	35 nS
E_K_	−58 mV
*Hyperpolarization activated current*	
G_h_	5.975 nS
E_h_	−17.7 mV
*Calcium current*	
G_Ca_	1.1 nS
[Ca^2+^]_o_	2,000 µM
*Calcium-dependent potassium current*	
G_Kc_	8.5 nS
E_K_	−58 mV
*Leak current*	
G_l_	0.5 nS
E_l_	−50 mV
*Gap junction*	
G_GJ-A2-BC_	200 nS
	
**ON-RGC**	
C	1.26 × 10^−10^ F
E_k_	−77 mV
E_na_	50 mV
E_leak_	−100 mV
g_leak_	3.1 × 10^−12^ S
G_syn_	250 × 10^−12^ S

**Table 3.** ON-CBC calcium buffer parameters

Calcium buffer	
F	9.649 × 10^4^
V_s_ (volume)	1.692 × 10^−14^ dm^3^
V_d_	7.356 × 10^−14^ dm^3^
D_Ca_	6 × 10^−8^ dm^2^ s^−1^
α_bl_	0.4 (s µM)^−1^
α_bh_	100 (s µM)^−1^
β_bl_	0.2 (s µM)^−1^
β_bh_	90 (s µM)^−1^
J_ex_	9 pA
J_ex2_	9.5 pA
Ca_min_	0.05 µM
S_sd_	4 × 10^−8^ dm^2^
d_sd_	5.9 × 10^−5^ dm

#### RGC model.

The RGC model is a simple spike-generating mechanism consisting of three ion channels responsible for a sodium current, an M-Type potassium current, and an A-Type potassium channel. In addition to these ion channels, there is synaptic current, which is modeled as a current that depends on the calcium current of the bipolar cell. Finally, a leak current was added as well. The sodium channel, M-Type potassium channel, and A-Type potassium channels and the leak current are described with the same equation used for the A_II_ ACs. The synaptic current is given by

$$I_{\mathrm{synapse}}=G_{\mathrm{synapse}}\cdot I_{\mathrm{CaONCB}}\cdot (V+35).$$

The membrane potential of the RGC is given by

$$C\frac{dV_{\mathrm{RGC}}}{\mathrm{dt}}=-\left(I_{\mathrm{Na}}+I_{M}+I_{A}+I_{\mathrm{leak}}+I_{\mathrm{synapse}}\right).$$

## Supplementary Material

Appendix 01 (PDF)

Movie S1. The activation time constant of INa, located on the IS, which is connected to the soma via a cable-like compartment, increases 10-fold from 0.01 to 0.1 ms. The movie shows the effect of these changes on the big oscillations in the soma. As can be seen in the beginning of the movie, the depolarizing phase of the oscillations becomes bigger and wider, and at some point a spikelet gets added to the oscillation just before the oscillation disappears.

Movie S2. The effect of the inactivation time constant is more pronounced than that of the activation time constant, while the relative change is smaller. The inactivation time constant changes from 0.5 to 4 ms. As can be seen in movie 2, the effect of modulating this parameter narrows the big oscillation in the soma while also increasing the amplitude. The number of spikelets reduces with an increase in the time constant, and the amplitude of the spikelets increases until there is just a blunted single spike left.

Movie S3. The conductance of the Na current (G_Na_) is the second most effective parameter to prevent the oscillations in the soma of the AII amacrine cell. At the beginning of the sweep a single spikelet disappears, after which the amplitude of the oscillation drops and the whole oscillation disappears in just 4 steps.

Movie S4. The effect of the modulation of *τ_h_2A__* the fast deactivation time constant of IKA, from 2 to 4, on the oscillations is very limited, you see the oscillation to slightly decrease in amplitude and widen the oscillation.

Movie S5. The influence of the modulation, from 2 to 4, of *τ_h_2A__* on the oscillation in the soma seems to increase the number of spikelets in the oscillation while having little influence on the width of the oscillation.

Movie S6. The parameter K_mAK_ controls the sensitivity of the voltage dependency of the activation of I_KA_. This parameter seems to prevent the oscillations the quickest. In the beginning of the sweep you can see a single spikelet disappear from the oscillation before the whole oscillation disappears at step 3.

Movie S7. The parameter f0, which controls the ratio between activation and deactivation of IKA, decreases from 0.96 in control to 0.9 in dopamine conditions. This mainly affects the amplitude of the oscillations and removes a spikelet at some potentials.

Movie S8. The gain of IKA in the soma of the AII amacrine cell has a large effect on the amplitude as well as the width of the oscillation, increasing both. By increasing the width, the number of spikelets on top of the oscillation also increases.

Movie S9. In the beginning of the sweep, the gain of IKA in the IS looks similar to that of the gain of IKA in the soma, increasing both the width and amplitude. However, after a few steps the whole oscillation disappears, this is due to the fact that the IS is not being able to produce spikes anymore, the spikes in the IS are essential for the big oscillation in the soma.

## Data Availability

Experimental data, analysis scripts, and model code supporting this study have been deposited in Zenodo (53).
