# Supplementary material for: Dopamine suppresses pathological retinal oscillations and enhances the signal-to-noise ratio
Source: Proc Natl Acad Sci U S A. 2026 Oct 6;123(41):e2535572123. doi: 10.1073/pnas.2535572123 (PMC13663775; doi:10.1073/pnas.2535572123)
Supplement: Supplementary file 1 [file pnas.2535572123.sapp.pdf]

# Supplementary Information

## Supplementary Figures 1-4

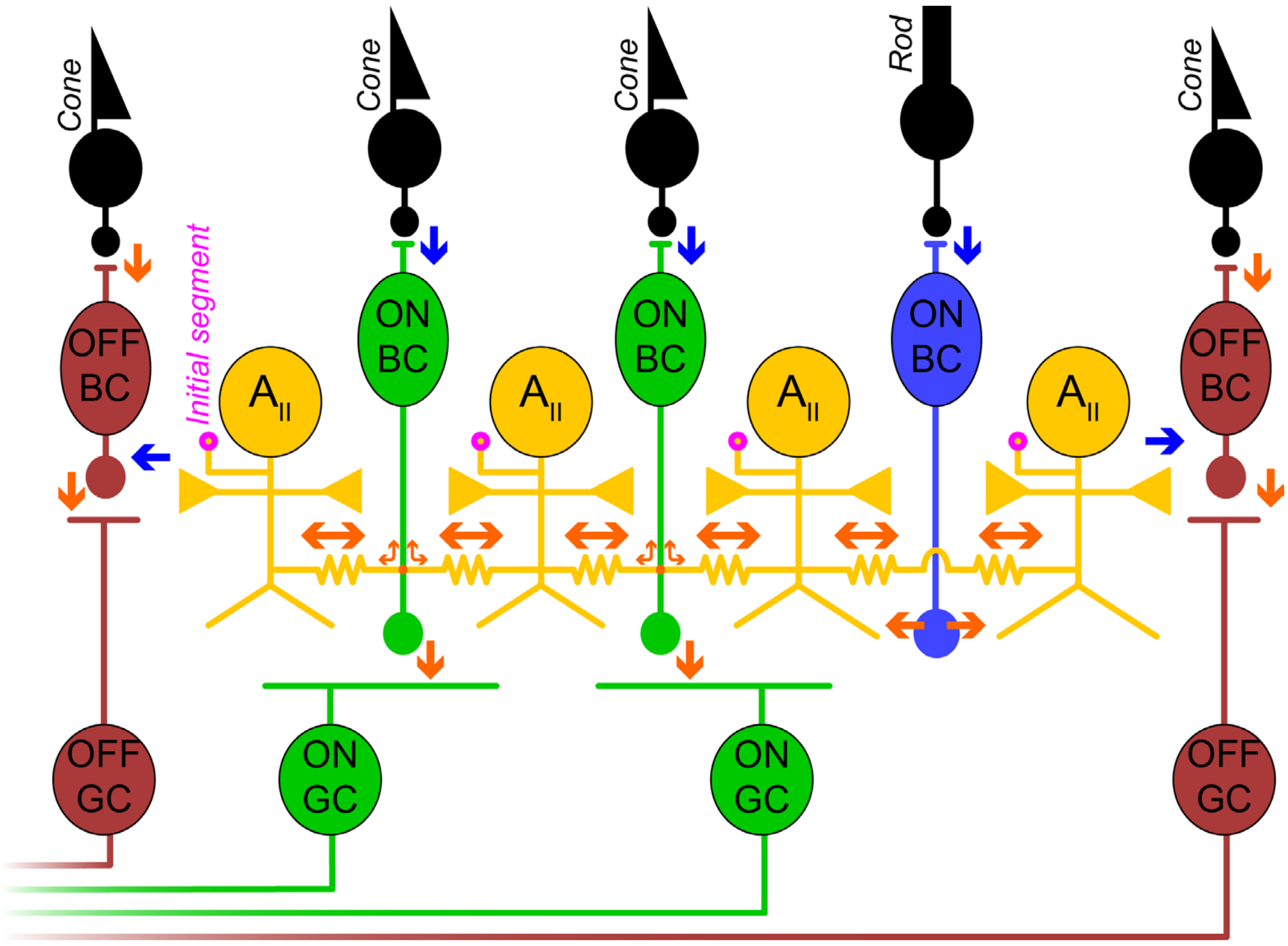


**Figure S1. Schematic of retinal microcircuit highlighting $A_{II}$ AC interconnectivity**. The diagram illustrates a retinal microcircuit comprising photoreceptors (cones and a rod), bipolar cells (OFF cone BCs in maroon, ON cone BCs in green, and a rod ON BC in blue), $A_{II}$ ACs (yellow), and ganglion cells (OFF GCs in maroon, ON GCs in green). The extensive interconnectivity of the $A_{II}$ ACs includes: (i) Direct electrical coupling between $A_{II}$ ACs via gap junctions (indicated by double-headed orange arrows), forming a network of these cells. (ii) Chemical synaptic input received from rod ON BCs (blue arrow from the rod ON BC to an $A_{II}$ AC). (iii) Electrical coupling with ON cone-BCs via gap junctions, allowing signals from the rod pathway to flow into the ON cone pathway. (iv) Chemical synaptic output to OFF cone BCs (blue arrows from $A_{II}$ ACs to OFF cone BCs), which relays rod-driven signals to the OFF pathway. The "Initial segment" of an $A_{II}$ AC, a site critical for its electrophysiological properties, is labeled in magenta. Blue arrows represent sign-inverting pathways, and red arrows represent sign-conserving pathways.

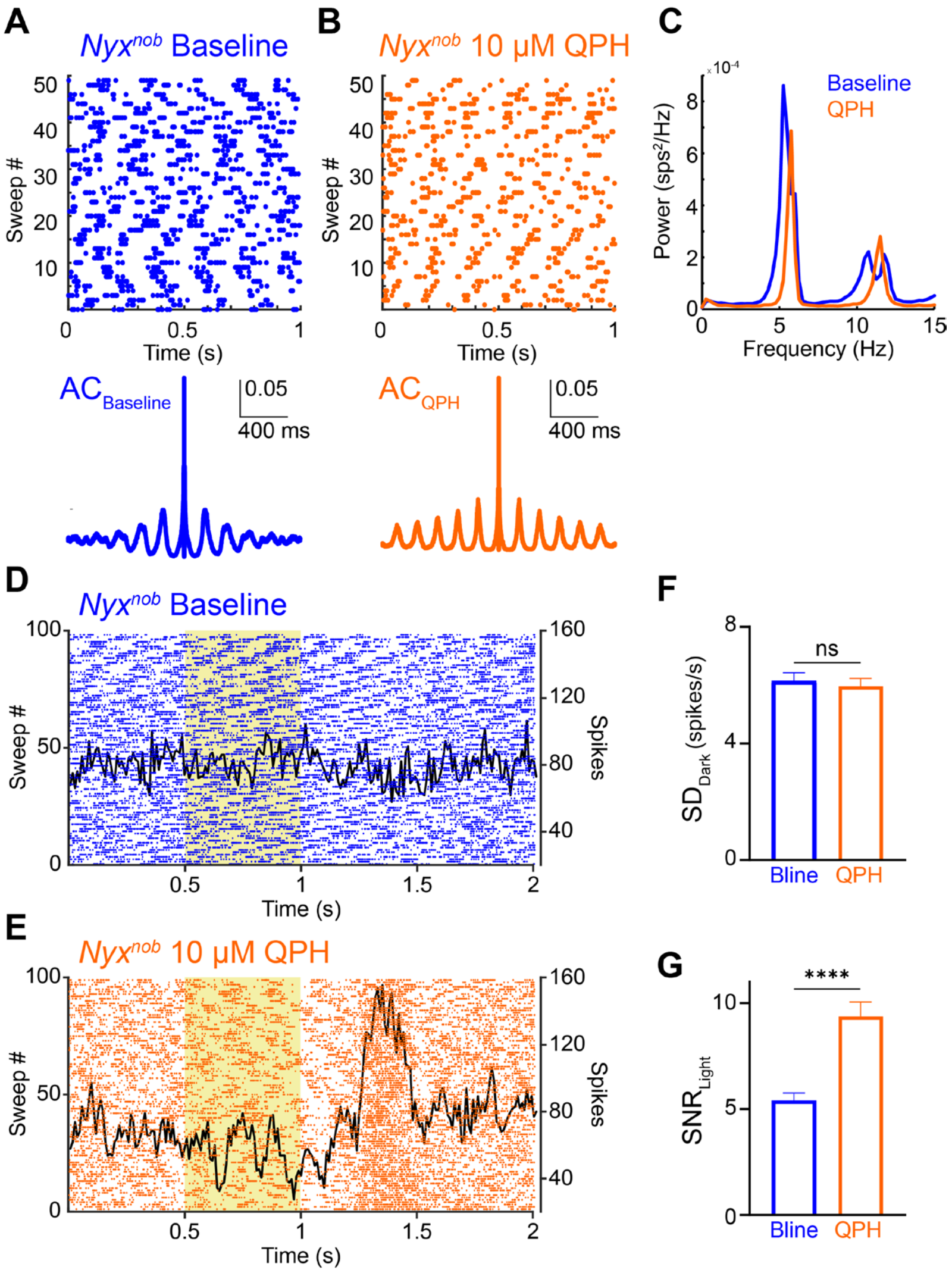


**Figure S2. Activation of D2-receptor failed to eliminate oscillations but enhanced the signal-to-noise ratio. A-B**, Spontaneous retinal ganglion cell (RGC) activity in the dark in an isolated light-adapted retina. Top: raster plot of a single RGC. Bottom: autocorrelation of the same RGC. **A**, Baseline *Nyx$^{nob}$* mouse retina showing robust oscillations. **B,** *Nyx$^{nob}$* retina with 10 µM quinpirole hydrochloride (QPH) application. **C**, Mean power spectral density of RGC activity from a representative cell under baseline conditions (blue) and with QPH (red). **D**, Example of a single RGC's response to light stimulation in a baseline *Nyx$^{nob}$* retina. Raster plot (blue). Overlaid corresponding peri-stimulus time histogram (PSTH, black). **E**, Same single RGC as in (**D**) but after application of 10 µM QPH (orange), illustrating increased peak light response. **F**, Bar graphs quantifying the mean standard deviation (SD) of baseline RGC firing rates (noise) in the dark period preceding light stimulation under baseline (blue) and 10 µM QPH (orange) conditions. SD: baseline (blue; mean 6.14, n = 122) vs QPH (orange: 5.96, n = 122; $P = 0.23$, Wilcoxon test). **G**, Bar graphs quantifying the mean signal-to-noise ratio (SNR) of RGC light responses for baseline (blue) and 10 µM QPH (orange) conditions. SNR: baseline (blue; mean 5.39, n = 122) vs QPH (orange: 9.38, n = 122; $P < 0.0001$, Wilcoxon test).

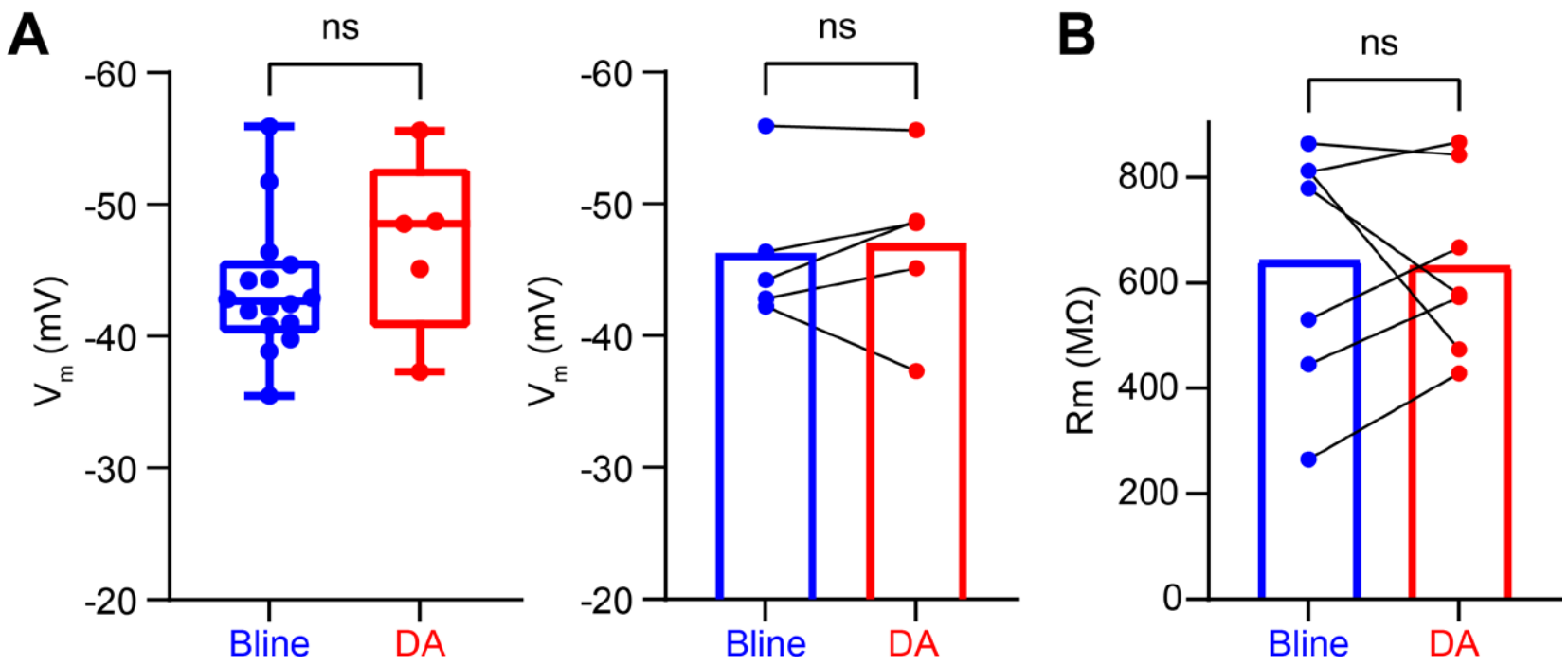


**Figure S3. Dopamine does not alter passive membrane properties. A**, Left: Box plots show the distribution of $V_m$ (mV) under baseline (Bline, blue) and 10 µM DA (red) conditions. $V_m$: baseline (blue; mean -43.5 mV, n = 16) vs DA (red; mean -47.1 mV, n = 5; *P* = 0.21, Mann-Whitney test). Right: Bar graphs with paired circles show data from individual cells. $V_m$: baseline (blue; mean -46.3 mV, n = 5) vs DA (red; mean -47.1 mV, n = 5; *P* = 0.81, Wilcoxon test) **B**, Bar graphs show the mean membrane resistance ($R_m$, MΩ) under baseline (Bline, blue) and 10 µM DA (red) conditions. Paired circles show data from individual cells. $R_m$: baseline (blue; mean 644.1 MΩ, n = 7) vs DA (red; mean 632.8 MΩ, n = 7; *P* > 0.999, Wilcoxon test).

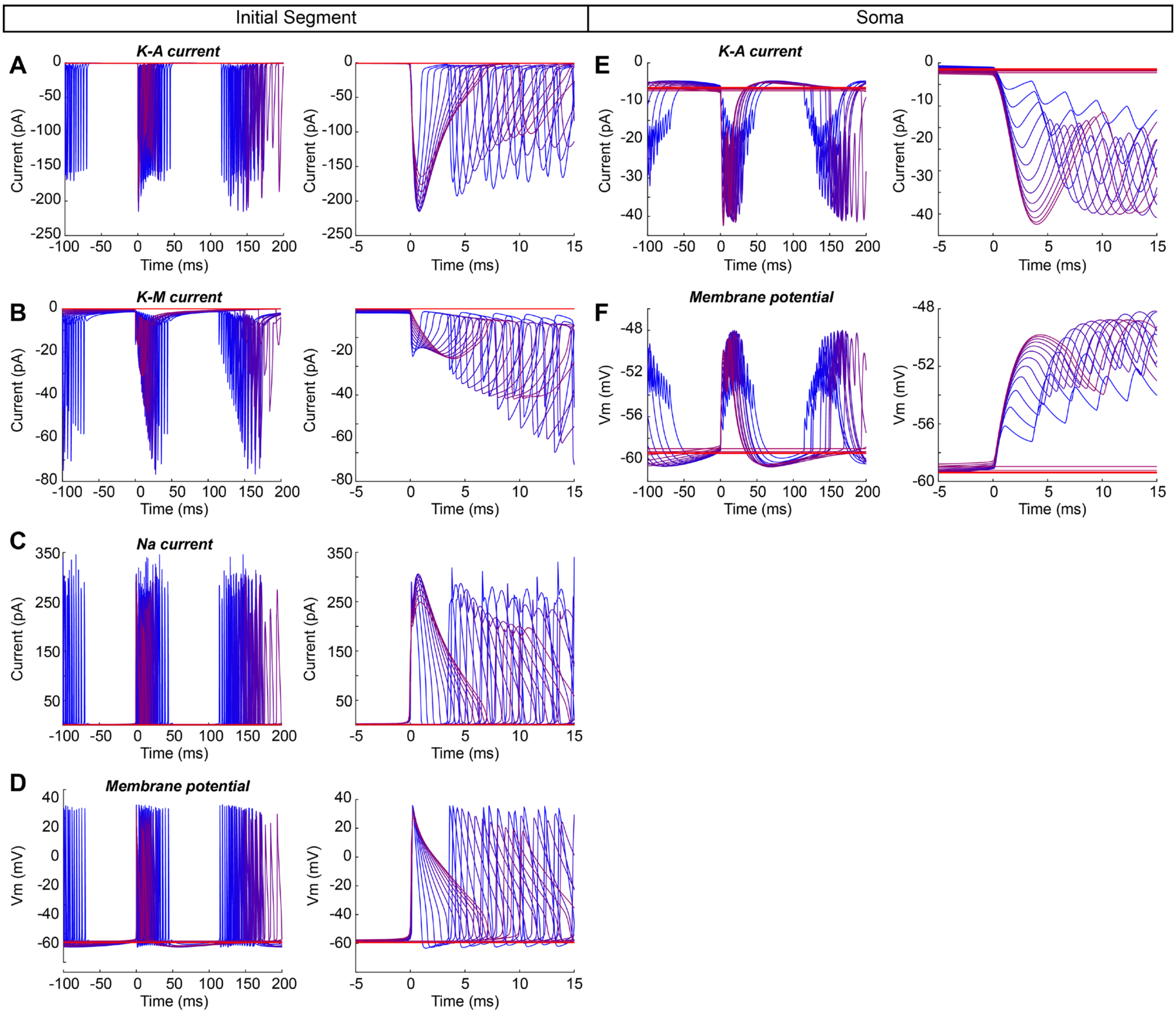


**Figure S4. Parameter sweep of the $A_{II}$ amacrine cell computational model from baseline to dopamine-modulated states.** Simulated currents and membrane potentials are shown for the initial segment compartment (**A–D**) and the somatic compartment (**E–F**). In all panels, trace colors represent a stepwise parameter sweep, the difference in parameters between baseline and dopamine condition were divided by 18 and increased stepwise from control -1 (blue, robust oscillatory bursting) to dopamine +1 (red, complete oscillation suppression) in 20 steps. Intermediate states are shown in transitioning shades of purple. For each lettered section, the left graph displays a broad time window (-100 to 200 ms) capturing the overall oscillatory rhythm and burst frequency, while the right graph provides a high-resolution view (-5 to 15 ms) of the current and voltage kinetics aligned to the onset of a single spikelet event. **A,** Simulated A-type potassium current ($I_{K\text{-}A}$) at the initial segment. **B,** Simulated M-type potassium current ($I_{K\text{-}M}$) at the initial segment. **C,** Simulated voltage-gated sodium current ($I_{Na}$) at the initial segment. **D,** Simulated membrane potential (Vm) at the initial segment. **E,** Simulated A-type potassium current ($_{IK\text{-}A}$) at the soma. **F,** Simulated membrane potential (Vm) at the soma.

## Supplementary Movies

During dopaminergic modulation of the $A_{II}$ amacrine cells, 9 parameters are modulated. Movies1-9 show the effect of variation of these parameters. We varied only one parameter at a time and displayed 20 steps. The second step was the control condition, and the $19^{th}$ step was the dopamine condition.

### $I_{Na}$

#### $\tau_{mNa}$ Movie 1

The activation time constant of $I_{Na}$, located on the IS, which is connected to the soma via a cable-like compartment, increases 10-fold from 0.01 to 0.1 ms. The movie shows the effect of these changes on the big oscillations in the soma. As can be seen in the beginning of the movie, the depolarizing phase of the oscillations becomes bigger and wider, and at some point a spikelet gets added to the oscillation just before the oscillation disappears.

#### $\tau_{hNa}$ Movie 2

The effect of the inactivation time constant is more pronounced than that of the activation time constant, while the relative change is smaller. The inactivation time constant changes from 0.5 to 4 ms. As can be seen in movie 2, the effect of modulating this parameter narrows the big oscillation in the soma while also increasing the amplitude. The number of spikelets reduces with an increase in the time constant, and the amplitude of the spikelets increases until there is just a blunted single spike left.

#### $G_{Na}$ Movie 3

The conductance of the Na current ($G_{Na}$) is the second most effective parameter to prevent the oscillations in the soma of the $A_{II}$ amacrine cell. At the beginning of the sweep a single spikelet disappears, after which the amplitude of the oscillation drops and the whole oscillation disappears in just 4 steps.

## $I_{K\text{-}A}$

### $\tau_{h_{2A}}$ Soma Movie 4

The effect of the modulation of $\tau_{h_{2A}}$, the fast deactivation time constant of $I_{KA}$, from 2 to 4, on the oscillations is very limited, you see the oscillation to slightly decrease in amplitude and widen the oscillation.

### $\tau_{h_{2A}}$ IS Movie 5

The influence of the modulation, from 2 to 4, of $\tau_{h_{2A}}$ on the oscillation in the soma seems to increase the number of spikelets in the oscillation while having little influence on the width of the oscillation.

### $K_{mAK}$ Movie 6

The parameter $K_{mAK}$ controls the sensitivity of the voltage dependency of the activation of $I_{KA}$. This parameter seems to prevent the oscillations the quickest. In the beginning of the sweep you can see a single spikelet disappear from the oscillation before the whole oscillation disappears at step 3.

### $f_0$ Movie 7

The parameter $f_0$, which controls the ratio between activation and deactivation of $I_{KA}$, decreases from 0.96 in control to 0.9 in dopamine conditions. This mainly affects the amplitude of the oscillations and removes a spikelet at some potentials.

### $G_{KA}$ Soma Movie 8

The gain of $I_{KA}$ in the soma of the $A_{II}$ amacrine cell has a large effect on the amplitude as well as the width of the oscillation, increasing both. By increasing the width, the number of spikelets on top of the oscillation also increases.

### $G_{KA}$ IS Movie 9

In the beginning of the sweep, the gain of $I_{KA}$ in the IS looks similar to that of the gain of $I_{KA}$ in the soma, increasing both the width and amplitude. However, after a few steps the whole oscillation disappears, this is due to the fact that the IS is not being able to produce spikes anymore, the spikes in the IS are essential for the big oscillation in the soma.
